# Differential roles of sleep spindles and sleep slow oscillations in memory consolidation

**DOI:** 10.1371/journal.pcbi.1006322

**Published:** 2018-07-09

**Authors:** Yina Wei, Giri P. Krishnan, Maxim Komarov, Maxim Bazhenov

**Affiliations:** Department of Medicine, University of California at San Diego, La Jolla, CA, United States of America; Radboud Universiteit Nijmegen, NETHERLANDS

## Abstract

Sleep plays an important role in the consolidation of recent memories. However, the cellular and synaptic mechanisms of consolidation during sleep remain poorly understood. In this study, using a realistic computational model of the thalamocortical network, we tested the role of Non-Rapid Eye Movement (NREM) sleep in memory consolidation. We found that sleep spindles (the hallmark of N2 stage sleep) and slow oscillations (the hallmark of N3 stage sleep) both promote replay of the spike sequences learned in the awake state and replay was localized at the trained network locations. Memory performance improved after a period of NREM sleep but not after the same time period in awake. When multiple memories were trained, the local nature of the spike sequence replay during spindles allowed replay of the distinct memory traces independently, while slow oscillations promoted competition that could prevent replay of the weak memories in a presence of the stronger memory traces. This could lead to extinction of the weak memories unless when sleep spindles (N2 sleep) preceded slow oscillations (N3 sleep), as observed during the natural sleep cycle. Our study presents a mechanistic explanation for the role of sleep rhythms in memory consolidation and proposes a testable hypothesis how the natural structure of sleep stages provides an optimal environment to consolidate memories.

## Author summary

We spend a third of our lives sleeping. During sleep, human and animal brains are decoupled from the external sensory input, however, brain activity remains high and varies across sleep stages. It is believed that one of the important sleep functions is the consolidation of recent memories to organize them for long-term storage. Two major sleep rhythms are observed during sleep–spindles (during stage 2) and slow oscillations (during stage 3)–and studies reported the importance of these rhythms in sleep-related memory consolidation. Nevertheless, the specific mechanisms of how these rhythms contribute to memory consolidation are poorly understood. Our new study predicts that sleep replay—repeatable sequences of neural cell firing, which are believed to be the neuronal substrate of memory consolidation—is organized within the patterns of the synchronous cell firing associated with these sleep rhythms. We found that for multiple interfering memories, sleep slow oscillations preferentially consolidate only the stronger memory traces, possibly leading to the weak memories extinction. In contrast, sleep spindles allow a slow but reliable consolidation of the multiple competing memories independently. Our study predicts how the basic structure of the natural sleep (where stage 2 precedes stage 3) provides an optimal environment for consolidation of multiple memories.

## Introduction

Sleep is believed to play an important role in consolidating of the recently learned knowledge [1–4]. During sleep-related consolidation, memories become increasingly enhanced and resistant to interference [5]. It was hypothesized that consolidation of memories during sleep occurs by reactivation of the neuron ensembles engaged during recent learning. Indeed, spike sequence replay was observed in the neocortex [6–8], following hippocampus-dependent tasks in coordination with replay in the hippocampus [6], and following hippocampus-independent task [9]. Sequence replay during sleep was proposed to be an important neural process involved in sleep-dependent memory consolidation [10].

The natural sleep cycle consists of several sleep stages: Stage 1 (N1), Stage 2 (N2), Stage 3 (N3) of non-rapid eye movement (NREM) sleep, and rapid eye movement (REM) sleep [11–13]. During NREM sleep, sleep spindles, 7–14 Hz brief bursts of rhythmic waves, are the hallmark of N2 sleep [14–16], while slow oscillations, characterized by repetitive (<1 Hz) Up and Down states in the cortical neurons [14, 17, 18], are mainly observed during N3 sleep (also referred as slow wave sleep). Although NREM sleep was shown to be particularly important for consolidating declarative (hippocampus-dependent) memories [19, 20], human studies suggest that NREM sleep may be also involved in the consolidation of the procedural (hippocampus-independent) memories, e.g. simple motor tasks [21], or finger-sequence tapping tasks [22, 23]. Indeed, selective deprivation of N2 sleep, but not a REM sleep, reduced memory improvement for rotor pursuit task [24]. Following a period of motor task learning, duration of NREM sleep [21] and the number of sleep spindles [25] increased. The amount of performance increase in finger tapping task correlated with the amount of NREM sleep [22], spindle density [26] and delta power [27]. Together studies suggest that NREM sleep is involved in the consolidation of the simple motor tasks, while REM sleep may become critical for learning the more complex memory tasks (see, e.g., [28]). A recent animal study [9] of consolidation of the procedural (skilled upper-limb) memory reported that reactivation of the neural activity was closely linked to the bursts of spindle activity and the waves of slow oscillation during NREM sleep. The role of NREM sleep oscillations in promoting consolidation is also supported by the studies where NREM oscillations were disrupted or generated optogenetically in the context of learning [29] or visual system plasticity [30] during NREM sleep. It was hypothesized that NREM sleep contributes to the consolidation of memories through the replay within the neocortex of the spike sequences associated with recent learning, however, the mechanisms behind sequence replay are poorly understood.

Here we used a biophysically realistic model of the thalamocortical network, implementing synaptic plasticity [31] and effects of neuromodulators [32], to explore basic mechanisms of the memory consolidation during NREM sleep. Our study predicts that sleep spindles and slow oscillations play unique and complementary roles in the consolidation of memories and that the natural sleep architecture, characterized by the well-defined sequence of sleep stages, is optimized to consolidate multiple mutually competing memories.

## Results

### Effect of sleep stages on memory recall performance

**Fig 1:**
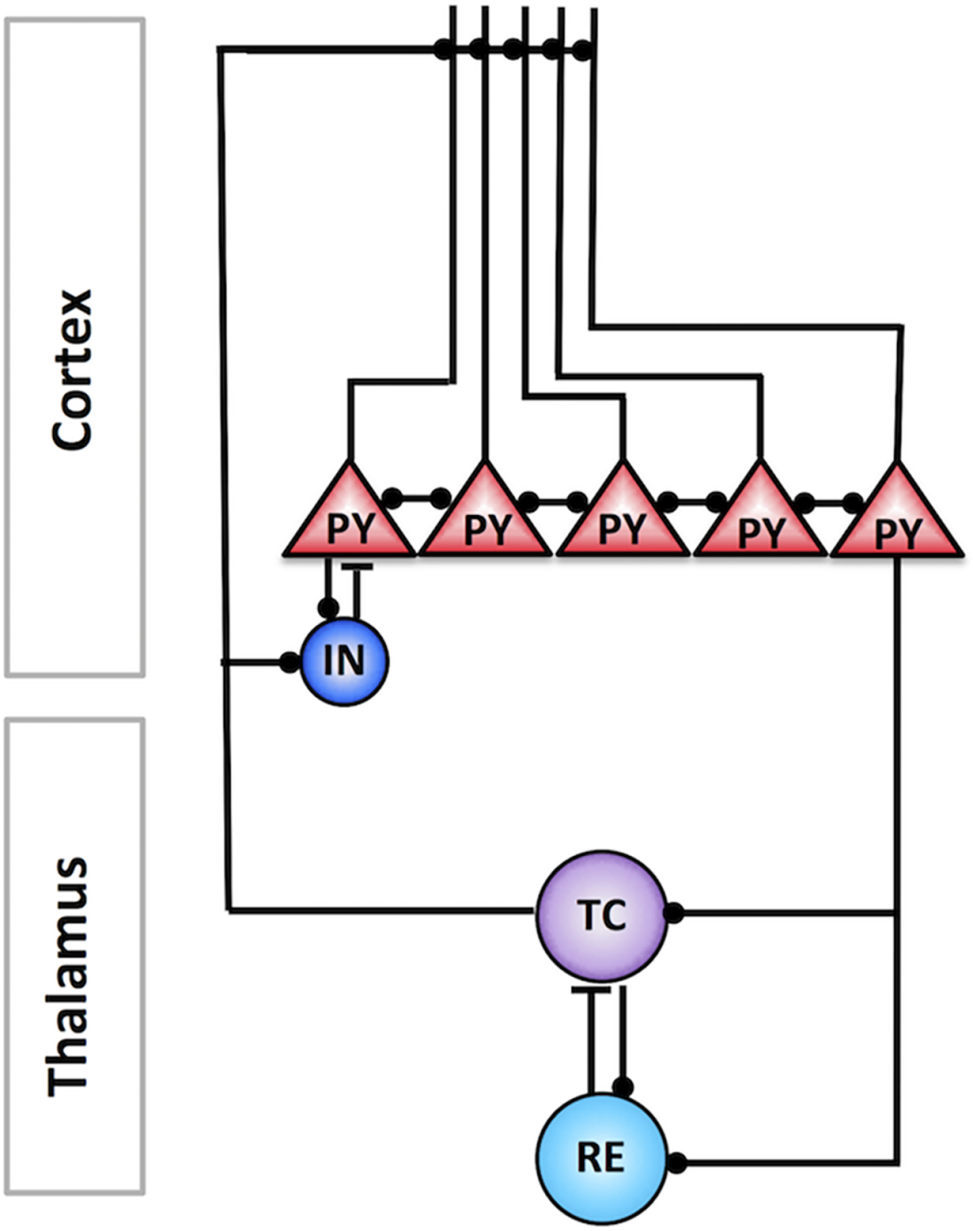
The schematic of the thalamocortical network model. The cortical layer was organized in a one-dimensional chain of pyramidal cells (PYs) and inhibitory neurons (INs). The thalamus model included thalamic relay (TC) and reticular thalamic (RE) neurons. Black filled circles and black bars represent excitatory and inhibitory connections between neurons, respectively.

**Fig 2:**
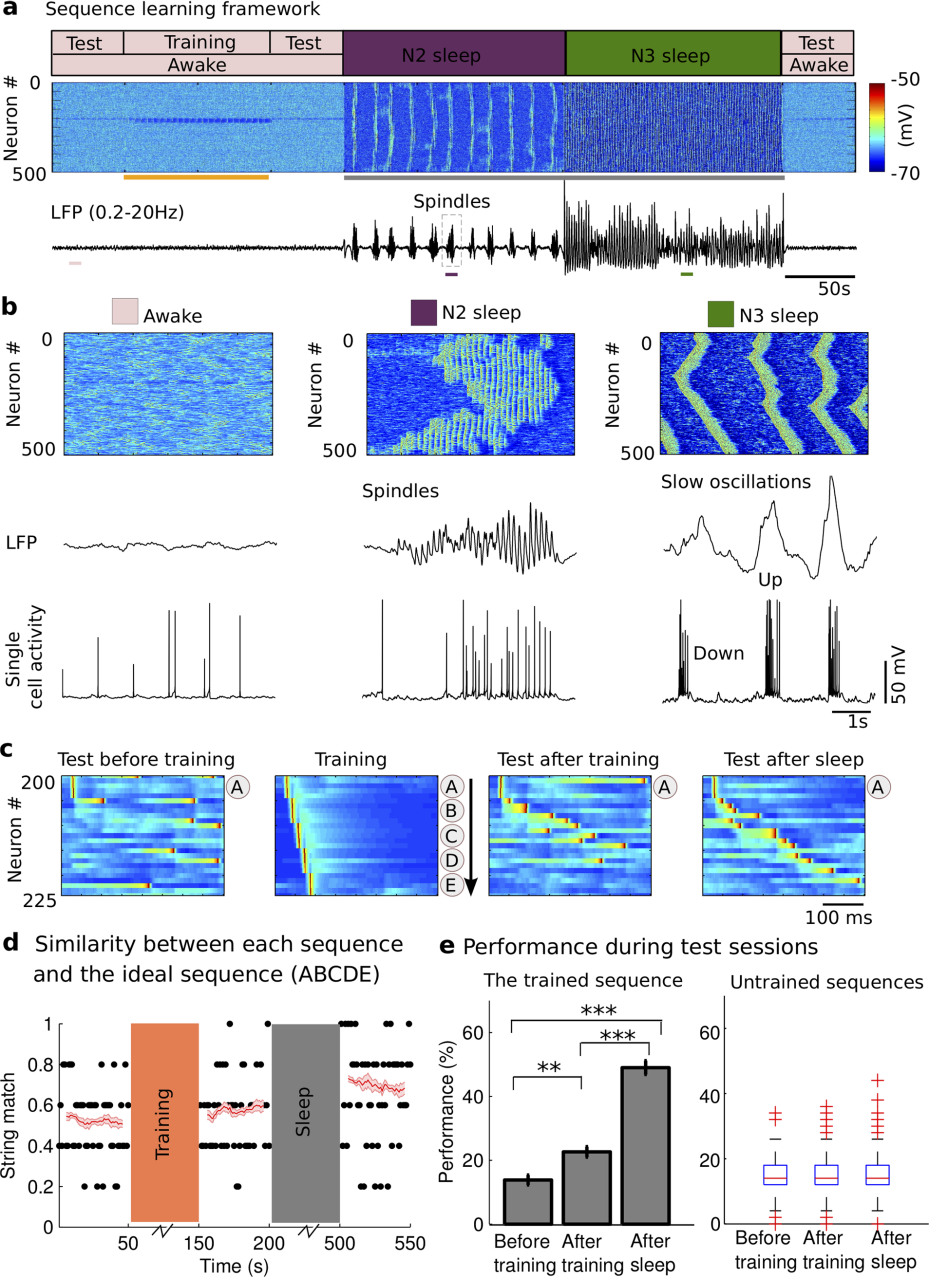
Network dynamics and sequence learning paradigm. **a**) The cortical network activity during transitions from awake state (pink block, *top*), to N2 sleep (purple block), to N3 sleep (dark green block) and back to the awake. Raster plot (*middle*) shows membrane voltages of cortical pyramidal cells. Broadband filtered local field potential (LFP, *bottom*) from the cortical population. The sequence was learned during the training period (orange bar). Grey bar represents the period of sleep. The performance was tested in three test sessions: before training, after training before sleep, and after sleep. **b**). The expanded view of characteristic spatiotemporal patterns (*top*), LFP (*middle*) and single cell activity of neuron #200 (*bottom*) during awake (*left*), N2 sleep (*middle*) and N3 sleep (*right*) from where pink, purple, dark green bars are shown in **a** (*bottom*). The spindle activity during N2 sleep revealed a typical waxing-waning pattern, consisted of 7–14 Hz brief bursts of rhythmic waves. The slow oscillations (<1Hz) during N3 sleep consisted of a typical Up and Down state transitions. **c**) The characteristic examples of a training session and three test sessions. The training included stimulating sequentially at groups A, B, C, D, and E. The test included stimulating only at group A (“pattern completion”). The sequence started at neuron #200. Each group included five neurons and it was stimulated for 10 ms. The delay between groups was 5 ms. **d**). The dot represents the string match between an ideal sequence (“ABCDE”) and each recalled sequence during test sessions for one trial. The value one represents a perfect match. The red line and the light red patch error bar represent mean and SEM of a moving average string match (window size = 10) over all trials (n = 10). **e**). The bar plot of the performance that was defined by the probability of recalled sequence with 80% similarity to the ideal sequence “ABCDE” (SM> = 0.8) during each test session. Error bars indicate standard error of the mean (SEM). For the boxplot in the right panel, the central mark indicates the median, and the bottom and top edges of the box indicate the 25th and 75th percentiles, respectively. *Left*: trained sequence; *Right*: untrained sequence tested at all other locations. * p<0.05, ** p<0.01, *** p<0.001.

We tested the role and the mechanisms of spike sequence replay for memory consolidation in the thalamocortical network model implementing awake, N2 and N3 sleep stages due to the variations in the level of neuromodulators [32]. The network model included thalamic relay (TC) and reticular (RE) neurons in the thalamus, as well as pyramidal neurons (PY) and inhibitory interneurons (IN) in the cortex (Fig 1, see Methods). Synaptic connections between cortical neurons were plastic, limited within [0, 200%] range, and controlled by STDP rules similar to our recent study [31]. We first simulated a basic sequence of sleep stages, including periods of awake, N2, N3 sleep and a second awake period following sleep (Fig 2A). Since our study is focused on understanding the role of the sleep rhythms observed during non-rapid eye movement (NREM) sleep–spindles and slow oscillations–in memory consolidation, we avoided modeling N1 sleep or rapid eye movement (REM) sleep. The awake state included one training session and three test sessions: before training, after training before sleep, and after sleep (Fig 2A). In the model, each network state had its own characteristic pattern of electrical activity as observed *in vivo* (Fig 2B). The neuronal activity during awake stage (Fig 2B, *left*) showed no specific spatiotemporal patterns and random fluctuations in the local field potentials (LFP), reflecting desynchronized cell firing. The N2 sleep (Fig 2B, *middle*) was characterized by the sleep spindle oscillations, consisted of 7–14 Hz brief bursts of rhythmic waves that lasted 0.5–3 seconds and recurred every 2–20 seconds [14–16], while N3 sleep (Fig 2B, *right*) was dominated by the slow oscillations (<1 Hz), characterized by repetitive Up and Down states in all cortical neurons [14, 17, 18]. We want to note that while we observed in the model the “waning” spindle activity at the beginning of Up states of slow waves in N3 [33], the overall spatiotemporal structure of the network activity during N3 sleep was dominated by the traveling slow waves and was very different from that during N2 sleep.

During the training session, the model was presented with multiple stimulation trials (delivered every 1s); each trial was a sequence of inputs to selected groups of cortical neurons (Fig 2C, *middle left*). Each group contained five neurons and was assigned a label (from A to E). By sequentially stimulating these five groups, the neuronal activity reflected sequential activation of the trained sequence, e.g., “ABCDE”. During test sessions (sequence recall), the model was only presented with the first input at group “A”. The characteristic examples of test sessions before training (Fig 2C, *left*), after training before sleep (Fig 2C, *middle right*), and after sleep (Fig 2C, *right*) showed a progressive increase of the correct sequence recall. To quantify memory recall performance, we used a string match (SM) measure (Fig 2D, black dots), which measures the similarity between each recalled sequence and the ideal sequence as trained, e.g. “ABCDE” (details in the method section). We found that there was an overall increase of SM after training, and then after period of sleep (Fig 2D, red line). We next calculated recall performance by measuring the success of a sequence recall—the percentage of the correct sequence recalls (with SM ≥ 0.8) for test stimulations (only group “A”) across multiple trials (Fig 2E). We observed a significant difference in performance of sequence recall among all three test sessions as determined by one-way ANOVA (F_2,27_ = 103.19, p = 2.26*10^−13^). The performance was significantly higher (p = 0.0056, Bonferroni corrections) after repetitive training (22.6%±1.63%) compared to the baseline performance before training (13.8%±1.53%), and was significantly higher after sleep (49.0%±2.18%) compared to that before sleep (p = 2.0174*10^−10^, Bonferroni corrections) (Fig 2E, *left*). Importantly, training had only minimal impact on the spatio-temporal patterns and the probability distribution of the Up state initiation sites across the network (S1 Fig). Indeed, we used in the model symmetric STDP rule that would generally increase synaptic weights in one direction but at the same time decrease weights in the opposite direction. As a result, the overall network excitability remained unchanged and characteristic properties of sleep and wake activity also remained throughout the entire sleep-wake simulation cycle.

We also tested performance of a “sequence” completion for the network locations outside the trained area. As above, the network was trained in the awake state to learn the sequence ABCDE (#200–224). Next, we applied test stimulation to the multiple random network sites that have not been trained, e.g. “A_1_” or “A_3_” (S2B Fig) and we were looking for sequence completion, analogues to ABCDE, initiating at these test locations (see Methods). Except for the trained region (#200–224), the performance of a sequence completion tested for random network locations showed no significant difference among all test sessions (Fig 2E, right), before or after the sleep, as determined by Kruskal-Wallis ANOVA test (F_2,957_ = 3.7, p = 0.157). In S2C Fig., we show the relative change of a sequence completion performance (after vs before the sleep) for individual network locations, and separately for two sequence directions, outside the trained region. We found no significant location or direction preferences and the histogram of the recall performance changes for all untrained sequences was centered at around 0 (S2D Fig). We conclude that only the trained area of the network revealed significant changes after the sleep, and it was no systematic changes at the other network locations.

### Synaptic mechanisms of memory consolidation during NREM sleep

**Fig 3:**
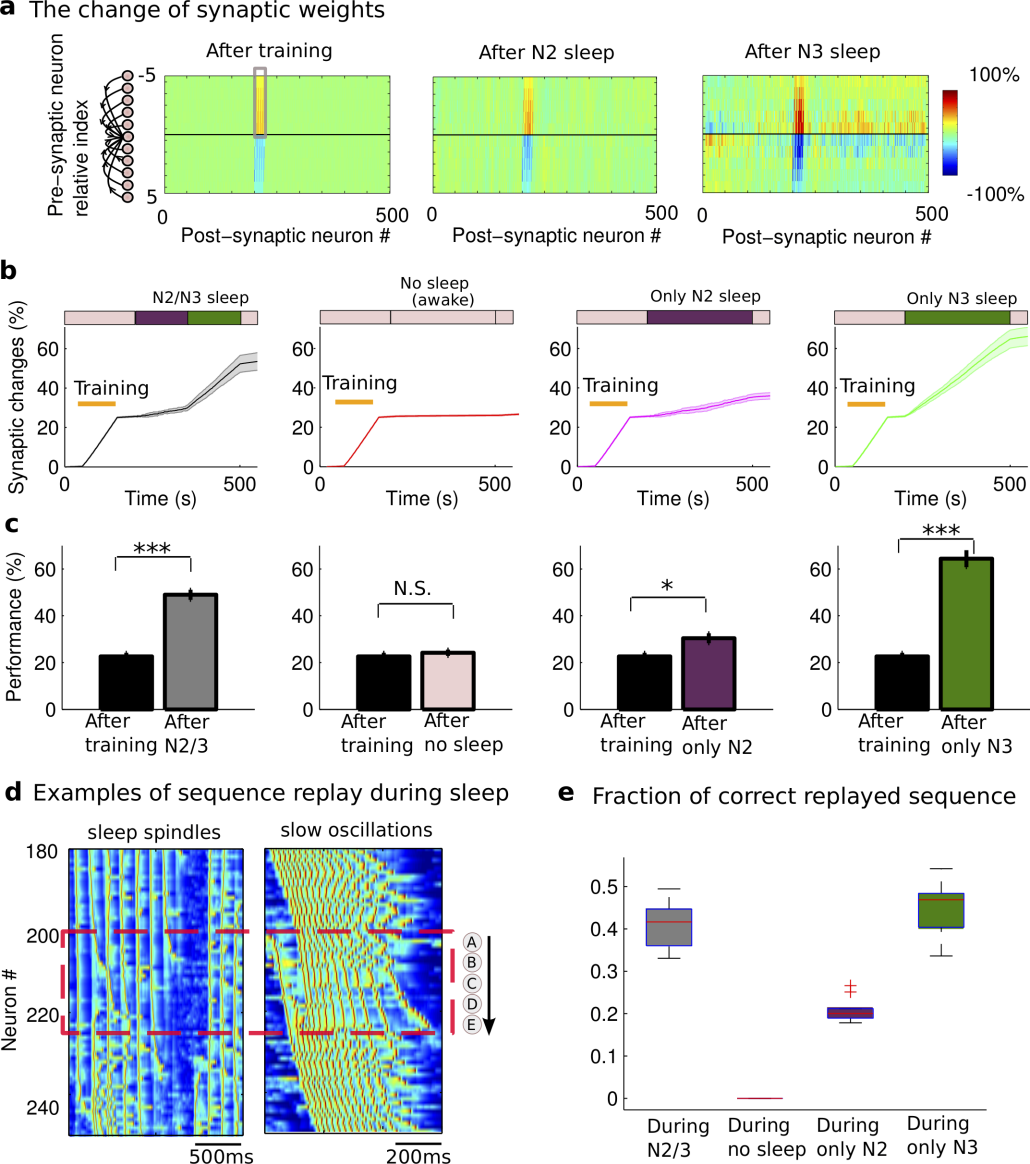
Spontaneous sequence replay mediates synaptic changes underlying memory consolidation during sleep. **a**) The change of synaptic weights relative to the initial values after training (*left*), N2 (*middle*) and N3 sleep (*right*). The synaptic weights between neurons in direction of sequence activation (grey box) were enhanced due to the sequence replay. **b**) The dynamics of the mean synaptic weights (grey box in **a**) shows the progressive increase in synaptic strength during normal N2+N3 sleep (*left*), only N2 sleep (*middle right*); only N3 sleep (*right*). Note the lack of synaptic changes when sleep was supplemented by awake state of the same duration (*middle left*). Orange bar represents training period. The blocks in the top summarize the protocol of each experiment: Pink block—awake, purple block—N2 sleep, dark green block—N3 sleep. The patch error bar represents standard deviation. **c**) The bar plots of performance during test sessions after training (before sleep) and after sleep in four different experimental conditions corresponding to **b**. Error bars indicate SEM. * p<0.05, ** p<0.01, *** p<0.001. N.S. represents no significant difference. **d**) Characteristic examples of sequence (“ABCDE”) replay during sleep spindles and slow oscillations. **e**) The fraction of correct replayed sequence (“ABCDE”) during four difference experimental protocols. For the boxplot in the right panel, the central mark indicates the median, and the bottom and top edges of the box indicate the 25th and 75th percentiles, respectively.

To reveal network changes underlying recall performance increase, we next analyzed the dynamics of synaptic weights between cortical neurons. During the initial training phase, the ordered firing of neurons led to potentiation of synapses between neurons in the order of the trained sequence, while the synapses corresponding to the opposite order of the learned sequence were depressed (Fig 3A, left). Importantly, the change in synaptic connections (Fig 3A, *left*, grey box) was observed during N2 (Fig 3A, *middle*) and continued in subsequent N3 sleep (Fig 3A, *right*). Overall, we found a progressive increase in synaptic weights that strengthened the trained sequence (Fig 3B, *left*) during sleep; this led to a significant enhancement of the recall performance after sleep (Fig 3C, *left*).

Although in this study we only systematically tested “linear” sequences (such as “ABCDE”), we found that the model predictions can be also extended to the case of more complex “nonlinear” sequences (e.g., ACBDE) (see Methods). In these simulations, to ensure that all the neurons are synaptically connected, we decreased the size of each activated neuronal group from 5 to 2 neurons. As with simple linear sequences, we observed a significant increase in the complex sequence (ACBDE) recall performance after the sleep compared to that before sleep (p = 4.33*10^−5^, one-way ANOVA, Bonferroni corrections) (S3 Fig). Note, that reducing group size made the net synaptic strength between any two groups weaker and, as a result, affected baseline performance, as well as performance after the training. Nevertheless, as long as any two neurons (within a sequence) that are expected to spike sequentially (e.g., AC) were synaptically connected (i.e., A→C), training of the complex sequence led to the corresponding synaptic changes and a significant increase in recall performance.

In order to identify the role of different sleep stages in memory consolidation, we next compared the change of synaptic weights and performance in four different conditions: 1) N2+N3 sleep (Fig 3B and 3C, *left*); 2) No sleep (Fig 3B and 3C, *middle left*); 3) only N2 sleep (Fig 3B and 3C, *middle right*); 4) only N3 sleep (Fig 3B and 3C, *right*). We found that recall performance of newly learned sequence was significantly enhanced after sleep (Fig 3C)—either only N2 (t(9) = -2.9351, p = 0.0166, two-sample t-test), only N3 (t(9) = -11.7468, p = 9.2315*10^−7^, two-sample t-test), or N2+N3 sleep (t(9) = -8.2644, p = 1.7056*10^−5^, two-sample t-test), but not after an equivalent awake period (t(9) = -0.6423, p = 0.5367, two-sample t-test; Fig 3C, *middle left*). Synaptic potentiation in the direction of sequence learning represented a basic mechanism of the recall performance increase in all sleep conditions (Fig 3B) and it was not significant during the equivalent awake period represented by the low level of background activity (Fig 3B, *middle left*).

### Role of sequence replay in memory consolidation

To reveal the neuronal mechanisms of synaptic reorganization during sleep, we analyzed the sequence reactivation during sleep of five groups of cortical neurons belonging to the sequence that was trained in awake (Fig 3D, in the dotted red box). We found that the trained sequence was reactivated spontaneously during spindles (Fig 3D, *left*) and slow oscillations (Fig 3D, *right*). The fraction of correct sequence replay during sleep (either N2+N3, or only N2, or only N3) was significantly higher compared to the equivalent awake period (p<0 for all comparisons, Mann-Whitney U test) (Fig 3E). We also observed a higher number of sequence replays during slow oscillation vs. spindles over the same period of sleep, which explains higher performance after N3 sleep alone vs. N2 sleep alone (compare Fig 3C, *middle right* and *right*). Finally, we found that the difference between direct and reverse sequence replays calculated for different network locations peaked at the location corresponding the trained sequence (S2E Fig.). This is consistent with earlier analysis that revealed no significant difference, before vs. after the sleep, in recall performance of a sequence completion tested for the random network locations (Fig 2E, right). Thus, we conclude that spontaneous emergence of the sequence replay during sleep led to potentiation of synapses corresponding to the trained sequence and resulted in performance improvement after the sleep. The replay was localized in the area corresponding to recent training. Both sleep spindles and sleep slow oscillations provided the spike timing structure that was necessary for successful sequence replay and memory consolidation.

To explore the role of specific characteristics of brain rhythms in memory consolidation, we increased the firing rate in the awake state from around 0.6 Hz (S4B Fig) to 1.7 Hz (S4C Fig) by increasing the mEPSP amplitude from 0.2 mV to 0.3 mV in the model. Although increasing awake firing activity increased the baseline performance (S4C Fig), because the high firing rate led to the higher occurrence of “chance” replays, there was no significant difference in the recall performance measured right after the training and after the subsequent period of awake without sleep (t(9) = -1.0986, p = 0.3005, two-sample t-test). This suggests that asynchronous firing in awake (even at a higher rate) lacks the structure necessary for reliable sequence replay of the trained spike sequences. Next, we reduced density of spindle events by reducing 10% potassium leak conductance of thalamic relay neurons (S5 Fig). When spindle density was reduced from around 5/min (S5B Fig) to 3/min (S5C Fig), the performance difference before vs after N2 sleep became not significant (t(9) = -2.0009, p = 0.0764, two-sample t-test). This indicates that high enough spindle density is necessary for consolidation. This result is interesting on its own as reduced the density of spindles is well-characterized EEG feature of the Schizophrenia and is also associated with cognitive deficiencies [34, 35]. Lastly, we have reduced the frequency of slow waves from around 0.7 Hz (S6B Fig) to around 0.3 Hz (S6C Fig) by reducing by 10% synaptic AMPA conductance strength during N3 sleep, which led to increasing duration of the Down states and decreasing duration of the Up states. Although the performance after sleep was still significantly higher compare to that before the sleep (t(9) = -8.9845, p = 8.6596*10^−6^, two-sample t-test) (S6C Fig), we observed a significant decrease in the overall performance improvement compared to the baseline model (t(9) = -7.2835, p = 4.6461*10^−5^, two-sample t-test). In both experiments, reduction in performance was a consequence of the reduced number of spindles/slow waves during a given sleep state duration. We concluded that the properties of spindles and slow oscillations, such as frequency of spindle events and duration and density of Up states, that depend on the well-defined biophysical mechanisms, make a significant impact on the spike sequence replay.

### Differences in the spatiotemporal dynamics of spindles and slow oscillations determine replay and consolidation properties

**Fig 4:**
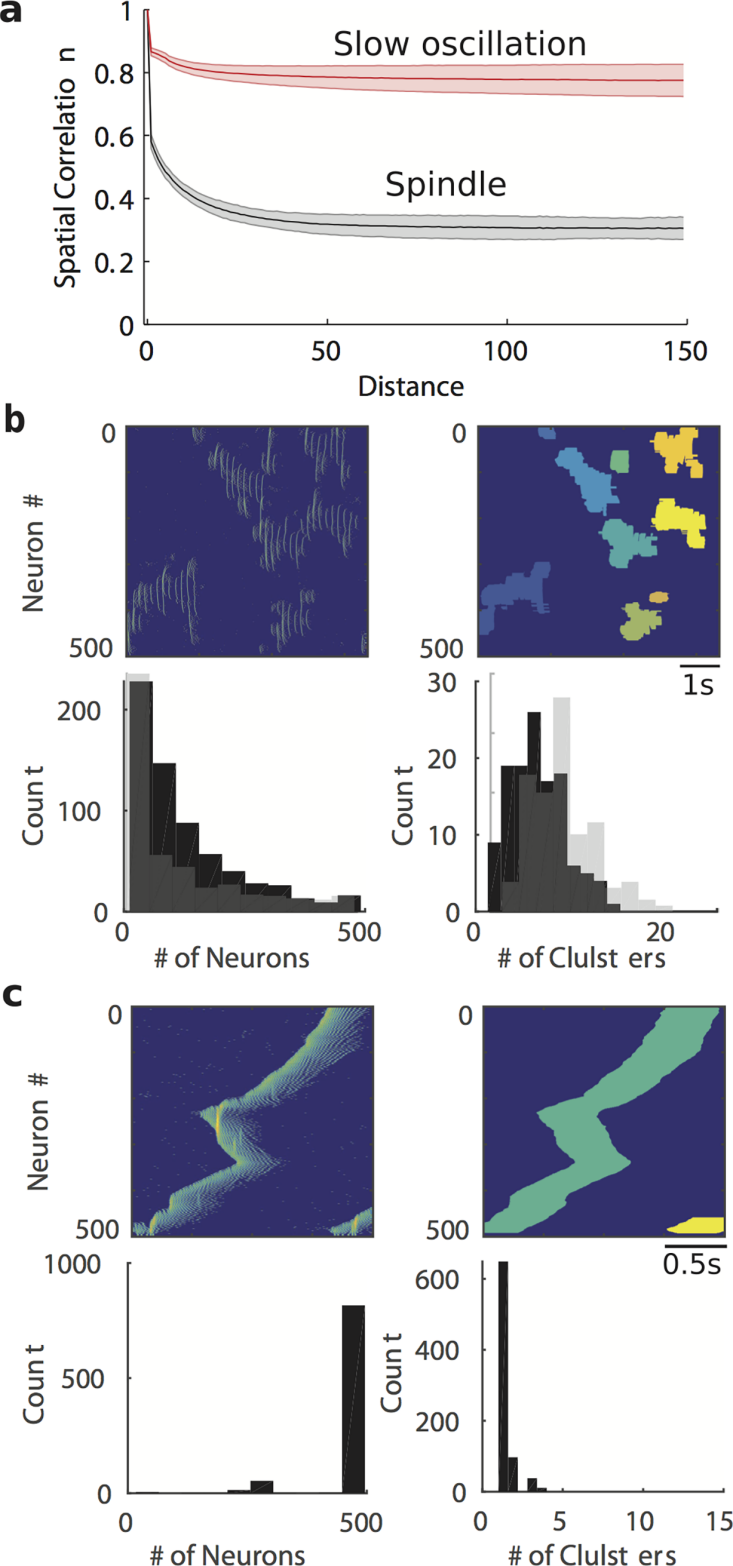
The differential spatiotemporal pattern of sleep spindles and slow oscillations. **a**) Spatial correlation between neurons at the different distance during sleep spindles and slow oscillations. The patch error bar represents standard deviation. **b**,**c**) An example of smoothed spike trains (*top left*) and the clustered region (*top right*), the histogram of neuron number (*bottom left*) that were identified within a cluster and the histogram of cluster numbers (*bottom right*) during spindle (**b**) and slow oscillations (**c**). The grey bar in **b**) is the histogram of temporally-cooccurring clusters that are monosynaptic connected during spindles.

Is the sequence replay during sleep spindles different from that during slow oscillations? While both spindles and slow oscillations may activate neurons within the STDP time window to enable plastic changes, the important difference seems to be in the overall spatiotemporal pattern. We first examined the cross-correlation of the Gaussian convoluted spike trains from the local groups of neurons (simulated local field potential) during spindles vs. slow oscillations. When the peak of the cross-correlation was plotted for varying distances between network sites, its value reduced with increasing distance during both spindles and slow oscillations (Fig 4A). However, the asymptotic level of the cross-correlation for remote network sites was significantly different between spindles (~0.35) and slow oscillation (~0.8) regimes (Fig 4A, black and red lines; t(149) = -116.1797, p = 4.5683*10–148, paired t-test), suggesting that activities of the cortical neurons during spindles are correlated only within relatively small regions, while during slow oscillations activity across the network is globally coordinated due to the nature of the traveling wave propagation.

We further examined the local versus global nature of spindles and slow oscillations using a spatiotemporal cluster analysis. We found that a typical single spindle event was built from many local clusters of neurons; while spiking was coordinated within each cluster, different clusters were semi-independent and initiated at the different network location. In contrast, the slow waves had a more organized global structure with each wave initiated at only one or few locations and traveling over the entire network. To explore this difference, we applied a clustering algorithm to count the number of neurons within each cluster for a slow wave or a spindle event (Fig 4B and 4C). While for slow oscillation a typical cluster included the entire population of neurons (500 cells in our network), for sleep spindles a cluster size was much smaller. We further extended this analysis by combining together all clusters co-occurring in time and separated by distance less than 5 neurons (radius of monosynaptic connection) and found very similar result (Fig 4B, bottom, gray bars). This analysis suggests that spindles may be better fitted to allow multiple memories to replay independently compare to the slow waves where multiple memories may have to compete within one large cluster defined by the global pattern of a slow wave propagation. In the next section, we will show that this difference makes a large impact on the replay and consolidation of “similar” memories competing for the overlapping or closely located ensembles of neurons.

### The role of slow oscillation in replay and consolidation of multiple sequences

**Fig 5:**
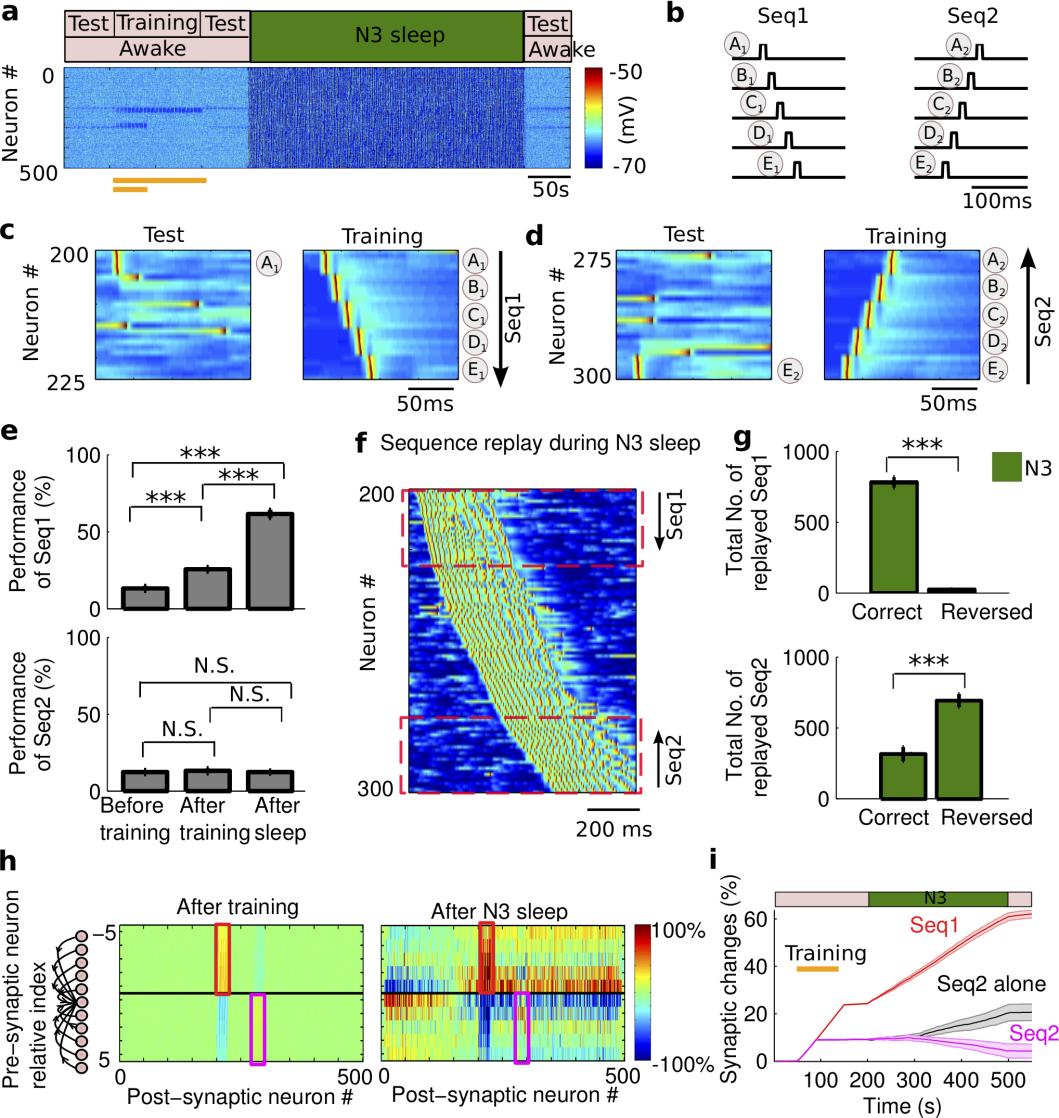
The role of slow oscillation during two-sequence learning. **a**) The model simulated transitions from awake to N3 sleep, and to awake again. Orange bar represents the duration of training of each sequence (*top*: Seq1; *bottom*: Seq2). **b**) A cartoon of the sequential network stimulation to generate two sequences. The duration of stimulation was 10ms for each group of neurons. The delay between subsequent stimuli of two groups was 5ms. Each group includes five neurons. **c**) A characteristic example of test and training of Seq1 (“A_1_B_1_C_1_D_1_E_1_”). The test was stimulating only at group A_1_. **d**) Test and training of Seq2 (“E_2_D_2_C_2_B_2_A_2_”). The test was stimulating only at group E_2_. The Seq1 and Seq2 started at neuron #200 and #300, respectively. **e**) The bar plots of performance for Seq1 and Seq2 during different test sessions. Error bars indicate SEM. **f**) A characteristic example of the sequences replay during slow oscillations. **g**) The bar plots of the total replayed Seq1 (*top*) and Seq2 (*bottom*) during N3 sleep in correct and reverse order. Error bars indicate SEM. The correct and reversed orders for Seq1 were “A_1_B_1_C_1_D_1_E_1_” and “E_1_d_1_C_1_B_1_A_1_”, respectively. The correct and reversed orders for Seq2 were “E_2_D_2_C_2_B_2_A_2_” and “A_2_B_2_C_2_D_2_E_2_”, respectively. **h)** The change of synaptic weights relative to the initial values after training (*left*) and after N3 sleep (*right*). Note that synaptic weights between neurons in the direction of Seq1 activation (red box) and Seq2 (magenta box) were both enhanced due to the training (*left*) but the effect decayed for Seq2 after N3 (*right*). **i)** The synaptic weights associated with Seq1 (red) were progressively increased during N3, while those associated with Seq2 (magenta) were decreased during N3 due to the interaction from the reactivation of Seq1. When Seq2 was trained alone (no interference) in the same experimental conditions, synaptic weights associated with Seq2 increased during N3 (black). The patch error bar represents standard deviation. * p<0.05, ** p<0.01, *** p<0.001. N.S. represents no significant difference.

**Fig 6:**
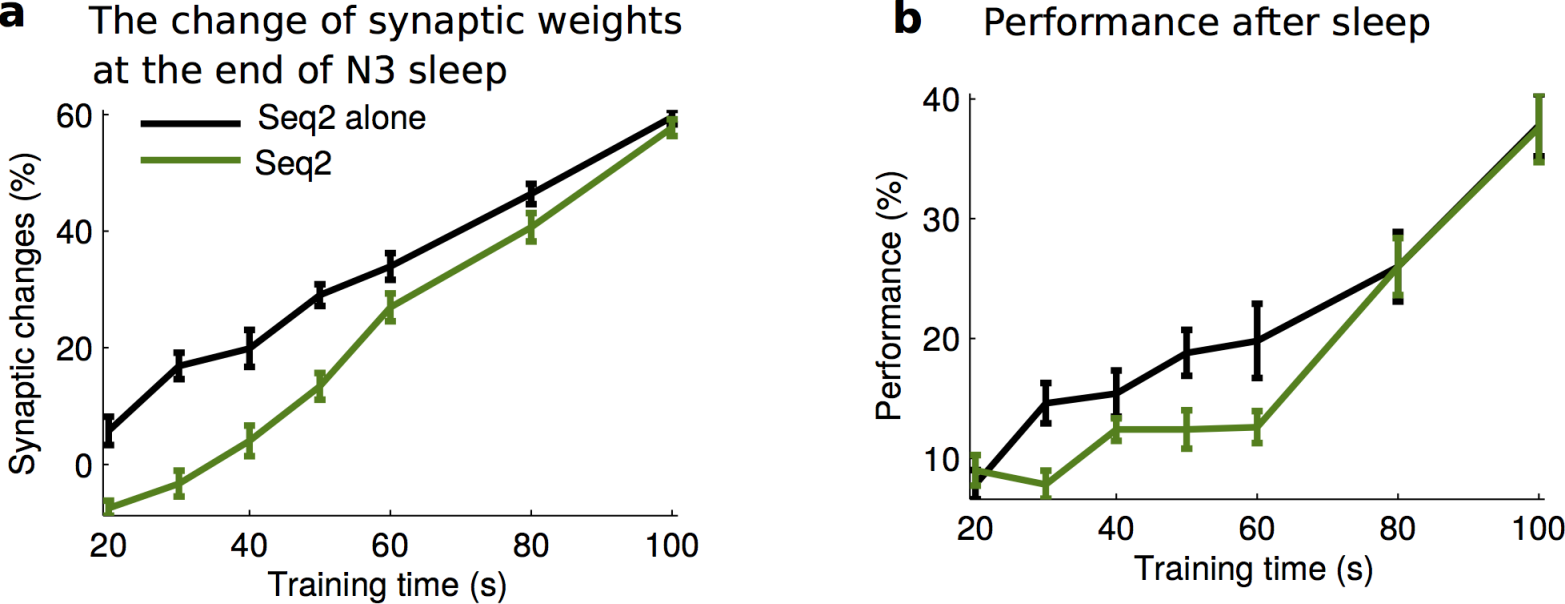
The effect of memory strength on the consolidation during slow oscillations. **a)** The dynamics of synaptic weights associated with Seq2 after N3 sleep for the different training duration (memory strength) of Seq2. The black line represents Seq2 trained alone. The dark green line indicates Seq2 trained along with the stronger Seq1. **b)** The change of Seq2 performance for the different training duration of Seq2. As the memory strength of Seq2 increased (longer training), the impact of interference on the synaptic weights and performance on Seq2 decreased. Error bars indicate SEM.

Human and animal brains can learn more than one motor sequences [36]. How do sleep rhythms coordinate multiple sequence replays? Here we show that spindles and slow oscillations play complimentary role in the consolidation of multiple sequences acquired in the awake state. First, we considered the “most challenging” (from competition perspective) case when the order of training of two sequences was opposite within the network topology and the neurons representing these sequences were relatively close in space (75 neurons distance between centers of the sequences), to explore the interaction between sequence replays during sleep. In these experiments, one sequence was trained longer (representing a strong memory) than another (representing a weak memory). Thus, the two sequences were trained by sequentially presenting stimuli at neuronal groups A1(#200–204), B1(#205–209), C1(#210–214), D1(#215–219), E1(#220–224) for Seq1, and groups E2(#300–296), D2(#295–291), C2(#290–286), B2(#285–281), A2(#280–276) for Seq2, respectively (Fig 5A, 5C and 5D). [We discuss effect of distance and sequence orientation, below in S7 Fig]. Seq1 “A_1_B_1_C_1_D_1_E_1_” was trained for 100s, representing a relatively “strong” memory. Seq2 “E_2_D_2_C_2_B_2_A_2_” was trained for 40s, representing a relatively “weak” memory (also see Fig 6 below for varying duration of a weak memory training). As before, recall performance for each sequence was measured based on the network responses by stimulating only first group of neurons in each sequence: group A_1_ (Fig 5C), or group E_2_ (Fig 5D).

In such conditions, when only N3 sleep was simulated (Fig 5A), we found a significant difference in the dynamics of the recall performance between strong (Seq1, F_2,27_ = 155.93, p = 1.47*10^−15^, one-way ANOVA) and weak (Seq2, F_2,27_ = 0.13, p = 0.8815, one-way ANOVA) memories. For Seq1, the performance was significantly increased (p = 5.16*10^−4^, Bonferroni corrections) after training (25.6%±1.5720%) over the baseline (13.2%±1.6653%), and further significantly improved (p = 1.8014*10^−15^, Bonferroni corrections) after the sleep (61.6%±2.6297%). In contrast, the performance of the weakly trained Seq2 (Fig 5E, *bottom*) was only slightly increased and was not significantly different from the baseline after initial training (13.2%±1.6111% vs. 12.4%±1.2579%, p = 1, Bonferroni corrections). Furthermore, it was not significantly improved (p = 1, Bonferroni corrections) after N3 sleep (12.4%±0.9333%). This change in performance was explained by the synaptic weight dynamics (Fig 5H). During the initial training phase in awake, the ordered firing of neurons led to synaptic potentiation for the synapses associated with Seq1 (Fig 5H, *left*, in the red box) and noticeable but less significant potentiation for the synapses associated with Seq2 (Fig 5H, *left*, in the magenta box). During the following N3 sleep, synaptic connections associated with the strong memory were further increased (Fig 5I, red line), however, in contrast, those associated with the weak memory were reduced (Fig 5I, magenta line). It is important to note that in the absence of the strong memory (Seq1), the weak memory (Seq2) alone would be enhanced during N3 sleep (Fig 5I, black line). Furthermore, a presence of a weak memory (Seq2) did not have significant effect on consolidation of the strong memory (Seq1), when compared to the case of Seq1 training alone (t(18) = 0.6225, p = 0.5414, two-sample t-test). These results can be explained by the interaction between two memories during slow oscillations: the strong memory was spontaneously reactivated in the correct order (Fig 5F) and the correct replay was significantly higher than the reversed replay (Fig 5G, *top*, t(18) = 20.477, p = 6.41*10^−14^, two-sample t-test), while the weak memory was replayed more in the reversed order than in the correct one (Fig 5G, *bottom*, t(18) = -5.48, p = 3.29*10^−5^, two-sample t-test). The later was happening because of the global pattern of slow waves propagation controlled by the network activity associated with the strong sequence. Therefore, during slow oscillation, the strong memory was preferentially replayed and enhanced while the replay of the weak memory, trained in the close proximity and the opposite direction to the strong one, was impeded.

To characterize how relative strength of the memory traces influences outcome of the sleep-dependent consolidation, we varied the training duration of the Seq2 with or without the presence of Seq1 (strong memory). For N3 sleep, synaptic changes associated with the Seq2 reversed the trend to decrease and started to increase as the training duration of Seq2 increased above ~50 sec (Fig 6A). However, the amount of this increase was significantly less than when Seq2 was present alone, unless Seq 2 was trained for the same duration as Seq 1 (Fig 6A, compare dark green and black lines; two-way ANOVA, F_1,126_ = 94.34, p = 0). Recall performance of Seq2 after sleep was also significantly reduced in the presence of Seq 1 for all durations except when Seq 2 was also trained strong (Fig 6B, compare dark green and black lines; two-way ANOVA, F_1,126_ = 8.56, p = 0.0041). The difference between two cases was getting smaller as the strength of Seq2 increased (Fig 6A and 6B). These results indicate that during slow oscillations, the presence of the strong memory trace, in close proximity to the cell population representing the weak memory, impede the replay of the weak memory. We found that, 40 sec duration of Seq 2 training (used in the simulations shown in Fig 5) represented a relative threshold when Seq 2 revealed no significant synaptic changes or performance improvement after the sleep. For durations of Seq 2 training less than the threshold, performance after sleep reduced below baseline and relative synaptic changes became negative. In contrast, for durations of Seq 2 training above the threshold, performance after sleep increased and relative synaptic changes were positive. Finally, the interference (negative impact of Seq 1 presence on Seq 2) was not observed at all when both memories were trained sufficiently strong before the sleep (e.g., trained for 100sec or longer). Thus, we conclude that there is a threshold for synaptic changes in our model that needs to be exceeded by initial training to allow replay and consolidation of the weak sequence in presence of the interference from another stronger memory.

### The role of sleep spindles in protecting weak sequence replay

**Fig 7:**
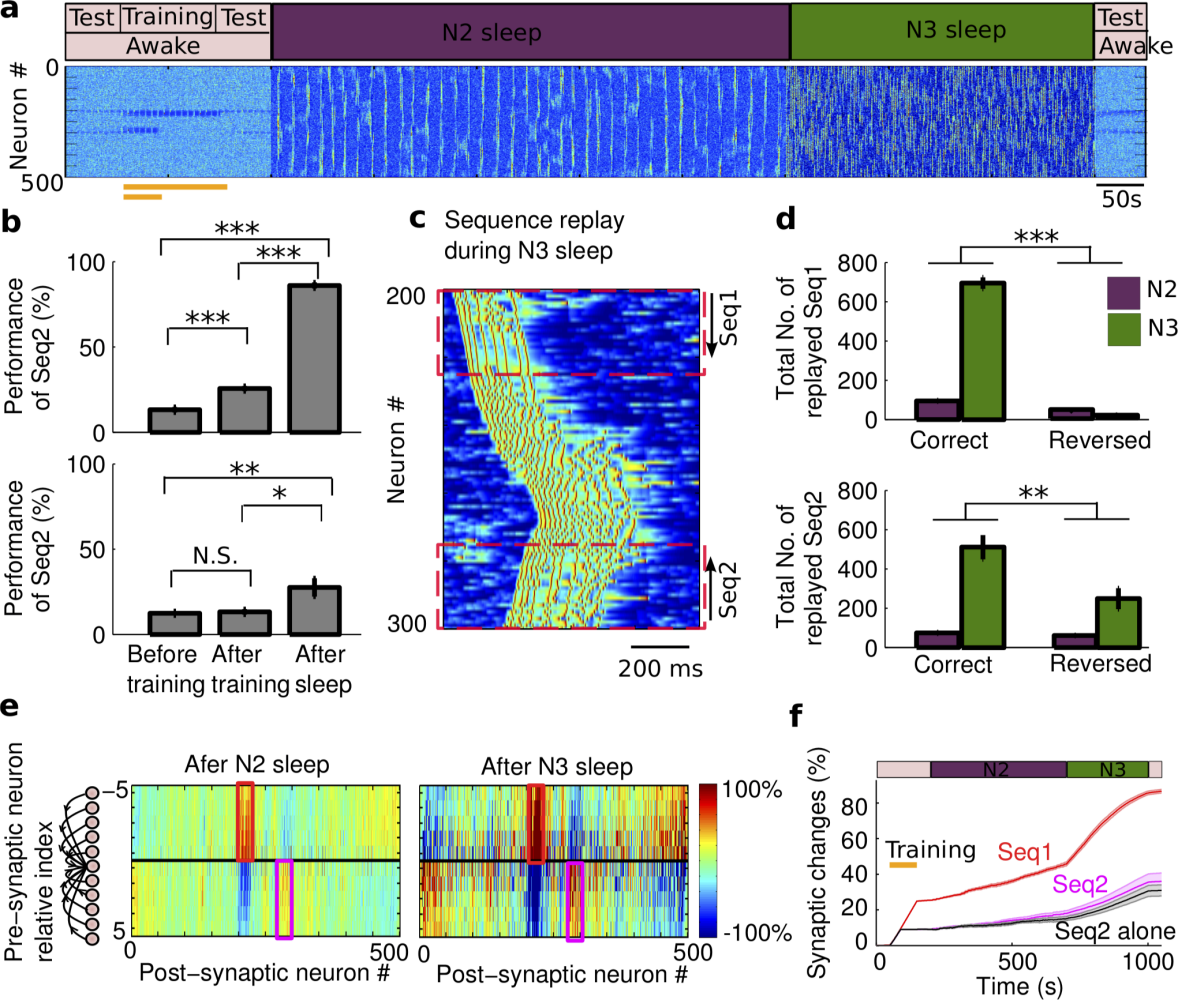
The role of sleep spindles during two-sequence learning. **a**) The model simulated transitions from awake to N2 sleep, to N3 sleep, and to awake again. Sequence training is the same as in Fig 3. **b**) The bar plots of performance for Seq1 and Seq2 during test sessions. Note significant increase in Seq2 performance after the sleep. Error bars indicate SEM. **c**) A characteristic example of sequence replay during slow oscillations. Note, that both Seq1 and Seq2 can be replayed during the same Up state of slow oscillation. **d**) The bar plots of the replay count for Seq1 and Seq2 during N2 (purple) and N3 (dark green) sleep. Error bars indicate SEM. Note that for both sequences number of correct order replays (“A_1_B_1_C_1_D_1_E_1_” for Seq1 and “E_2_D_2_C_2_B_2_A_2_” for Seq2) was higher than the number of reversed order replays. **e)** The change of synaptic weights relative to the initial values after N2 (*right*) and after subsequent N3 sleep (*left*). The synaptic change after training is the same as in **4j**). The enough amount of sleep spindles enhanced synaptic connections associated with both sequences independently. **f**). The progressive increase in synaptic weights associated with Seq1 (red), Seq2 (magenta), and Seq2 alone (black). The patch error bar represents standard deviation. * p<0.05, ** p<0.01, *** p<0.001. N.S. represents no significant difference.

We next tested the model with a sleep pattern similar to that of a natural sleep, where N3 was preceded by a period of N2 sleep (Fig 7A). In this sleep conditions, the overall performance of both Seq1 (F_2,27_ = 527.81, p = 2.28*10^−22^, one-way ANOVA) and Seq2 (F_2,27_ = 6.57, p = 0.0047, one-way ANOVA) were enhanced following the sleep period. Post hoc analysis for Seq1 (Fig 7B, *top*) indicated that performance was significantly increased (p = 8.1761*10^−20^, Bonferroni corrections) after sleep (86%±1.8379%) compared to that before sleep (25.6%±1.5720%). For Seq2, the recall performance (Fig 7B, *bottom*) also became significantly increased after sleep (27.6%±5.4062% vs. 13.2%±1.6111%, p = 0.01, Bonferroni corrections). In the presence of 500s N2 sleep preceding N3 sleep, we observed that both memories were reactivated more often in the correct order than in the reversed order during both N2 and N3 sleep (Fig 7C and 7D; Seq1: t(18) = 23.6913,p = 5.0806*10^−15^; Seq2: t(18) = 3.2747,p = 0.0042, two-sample t-test). This led to a progressive increase in the synaptic weights associated with both sequences (Fig 7E). The critical contribution of N2 sleep in memory consolidation was that during spindles synaptic weights representing correct order of firing increased both for the weak and strong memories (Fig 7F). This brought synaptic weights associated with Seq2 above the threshold, as described in the previous section, so the Seq2 became stronger enough and resistant to further interference from the reactivation of Seq1 during the N3 sleep.

### Effects of training duration, location and sequence orientation in multiple sequences replay

**Fig 8:**
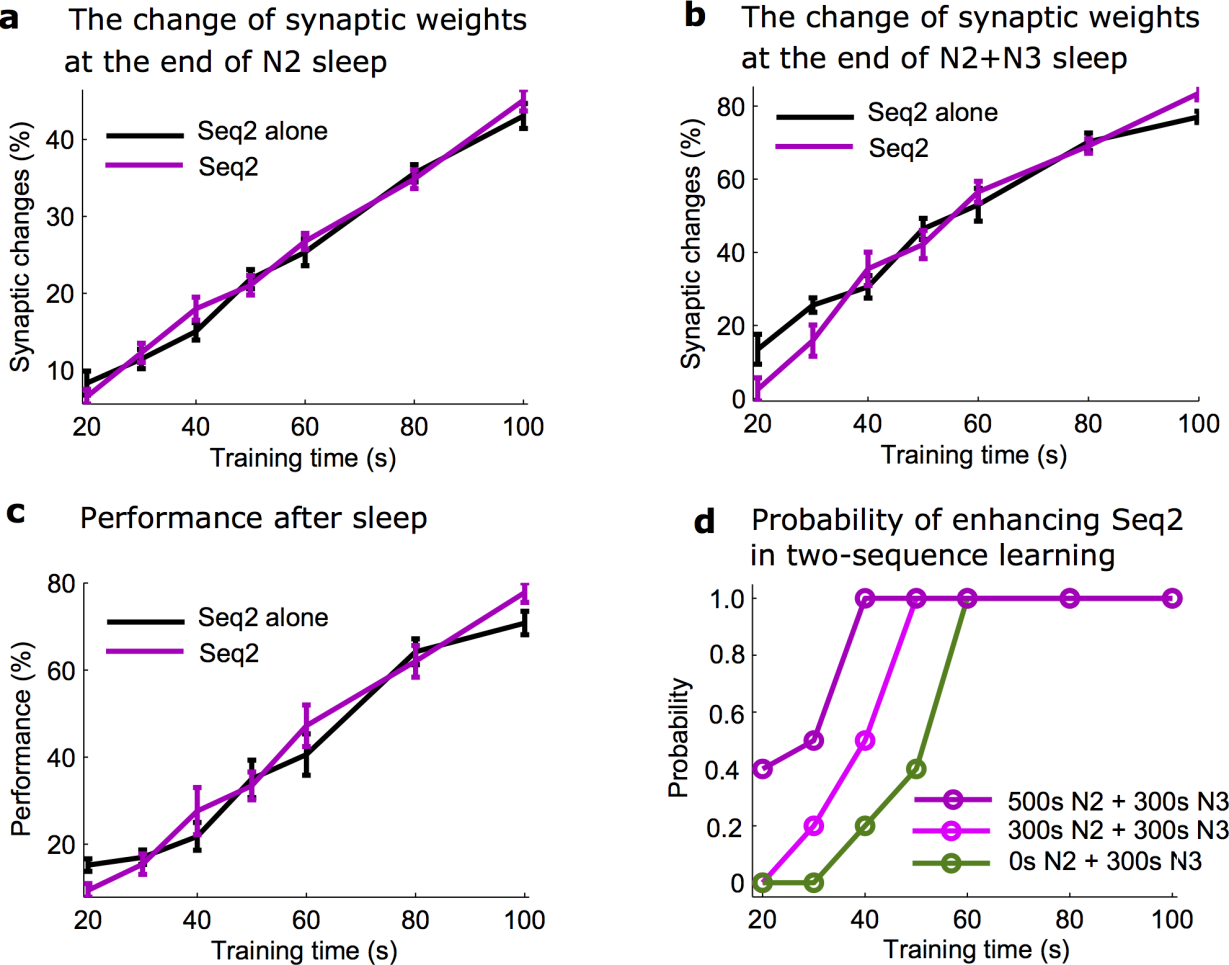
Effect of memory strength on the consolidation during normal N2+N3 sleep. **a,b)** The change of synaptic weights associated with Seq2 after N2 (**a**) and following N3 (**b**) sleep for the different training duration (memory strength) of Seq2. Importantly, after N2 sleep there is no difference in synaptic changes between Seq2 trained along with the stronger Seq1 and Seq2 trained alone. **c)** The change of Seq2 recall performance after N2+N3 sleep for the different training duration of Seq2. **d**) Probability across trials of synaptic weights increase for Seq2, when trained along with Seq1, for different duration of N2 sleep. Error bars indicate SEM.

N2 sleep supported the consolidation of the weaker memory for the varying strength (training duration) of Seq2 (Fig 8A, black and purple line, two-way ANOVA, F_1,126_ = 0.6, p = 0.4393), except when the Seq2 was extremely weak and then sleep spindle activity was not able to support Seq2 replay. We found that given enough of the spindle activity early in the sleep cycle, synaptic weights associated with Seq2 enhanced sufficiently to allow for the further increase during the subsequent N3 sleep. Overall, there was no significant difference in the change of synaptic weights associated with Seq2 between two groups (with or without the presence of Seq1) after the full period of sleep (N2+N3) in this condition (Fig 8B, two-way ANOVA, F_1,126_ = 0.84, p = 0.3598). The performance of Seq2 recall after the full period of sleep also showed no significant difference whether Seq1 was present or Seq2 was trained alone (Fig 8C, two-way ANOVA, F_1,126_ = 0.42, p = 0.5192).

We observed variability in the outcome of the Seq2 replay across individual trials, which was particularly high when Seq2 was weak (e.g., trained for 40s or less). Therefore, we next examined the probability (across trials) of improving the performance of Seq2 in the presence of Seq1. Successful consolidation was defined as a trend of synaptic weights to increase during last 100 sec of sleep. As expected, increasing duration of initial Seq2 training, increased a probability of consolidation which saturated at 100% for experiments with training duration exceeding ~60 sec. Importantly, as the duration of N2 sleep increased, the probability of successful Seq2 consolidation also increased, shifting probability curves to the left (Fig 8D). Thus, we conclude that sufficient amount of sleep spindles is necessary for the successful outcome of consolidation in experiments with a weak memory when other memories were imprinted in the same network (Fig 8D).

Lastly, to investigate how outcome of the memory consolidation during sleep depends on the distance between sequences and the spatial orientation of training, we varied the location and direction of Seq2 training while keeping Seq 1 fixed. To characterize the interference, we calculated the integral synaptic difference (see Methods) associated with Seq2 consolidation in simulations with and without the presence of Seq1 (S7 Fig). After N3 sleep, synaptic changes associated with Seq2 decreased (compare to the case of Seq 2 alone) when Seq 2 had the opposite direction of Seq1 and increased when it had the same direction of training as Seq1 (S7A Fig, compare solid and dotted lines; two-way ANOVA, p = 1.6667e-26). We also found that there was no significant difference in the amount of synaptic changes for Seq 2 for different spatial distances between two sequences (S7A Fig, two-way ANOVA, p = 0.3885). This suggests that the spatial orientation of the weak sequence in respect to the strong one determines whether it would be weakened or strengthened during slow oscillations. Specifically, for the case of the sequences with the same orientation, the presence of the stronger Seq 1 facilitated replay and consolidation of the weaker Seq 2. In contrast, after N2 sleep, analysis of synaptic weights changes associated with Seq2 revealed no significant difference for different spatial orientations or distances (S7B Fig, two-way ANOVA, p = 0.5376). This is consistent with our previous findings that spindles allow replay of both weak and strong memory traces independently, leading to reduced interference between memories trained in the opposite direction but also minimizing co-facilitation of replays for sequences trained in the same direction.

## Discussion

In this study, using a realistic computational model of the thalamocortical network implementing sleep stages [32] and sleep replay [31], we found that sleep spindles (7–14 Hz brief bursts of rhythmic waves) and sleep slow oscillations (<1 Hz rhythmic oscillations between Up and Down states of the thalamocortical network) both provide spatiotemporal structure of the cortical neurons’ firing that promotes spike sequence replay and organizes neuronal spiking in a way optimal for STDP to drive synaptic consolidation. The synaptic replay was localized in network space at the network locations corresponding to awake training. Importantly, sleep spindles allowed independent and simultaneous replay of multiple memories, even when these memories were competing for the same or similar ensembles of neurons. In contrast, sleep slow oscillation favored consolidation of the strong memories and could lead to the reverse replay and potentially to the extinction of the weak memories. Taking into account that slow oscillation allowed the faster rate of synaptic changes, our study predicts that a sequence of sleep stages N2 → N3, as observed during natural sleep in animals and humans, provides an optimal environment to reduce the interaction between memories during sleep replay and to maximize the efficiency of consolidation.

### Mechanisms of spontaneous sequence replay

Synaptic plasticity is believed to be the cellular mechanism of learning and memory in the brain. A large body of studies supports the idea that the spiking sequences of cortical neurons evoked by awake learning are replayed during sleep, leading to consolidation of memory [6–9]. In our new study, we focused on consolidation of the hippocampus-independent procedural memory traces, and we found that the sequences of the cortical neurons’ firing trained in awake, replayed spontaneously during NREM sleep. In N2 sleep replay occurred during spindle events and was phase locked to the spindle oscillations, while in N3 it involved bursts of the cortical cells firing during Up states of slow oscillations, consistent with the recent experimental findings [9]. We found no significant performance gain after an equivalent awake period, consistent with previous data [22, 37–39]. We need to mention, however, that imposing a background synchronized activity, such as e.g., alpha rhythm in the quiet awake [40], could potentially lead to replay and consolidation. However, the study of consolidation in awake would go beyond the scope of this paper that is focused on the role of the NREM sleep rhythms—spindles and slow oscillations—in memory consolidation.

Previous computational studies of the role of synaptic plasticity during sleep [41–43] mainly focused on the global synaptic weights dynamics to investigate synaptic homeostasis [44]. Our study did not reveal global synaptic weights downscaling as predicted by the synaptic homeostasis hypothesis [44], and we report an increase during sleep of the synaptic weights relevant to the recent learning [45]. We should note, however, that we used a symmetric STDP rule [42] and the model dynamics avoided high synchrony states, which may explain the global trend of synaptic weights dynamics in the model. In addition, neuromodulators have been shown to have a distinctive influence on STDP rules [46, 47]. Although we adjusted the amplitude of the STDP changes based on the level of the ACh during different sleep and wake stages, a more detailed neuromodulator-dependent STDP model should be explored in the future studies.

Although we focused on the hippocampus-independent memory replay (such as procedural memory) and thus our model does not include hippocampus, our results can be generalized to predict the role of NREM sleep in the consolidation of the hippocampus-dependent (such as declarative) memories. Hippocampal cell assembles reactivated during NREM sleep [48, 49] and spike sequence replay was reported to occur simultaneously in both hippocampus and neocortex [6] and it coincided with the hippocampal sharp-wave ripples (SWR) [50, 51]. Hippocampal outflow during SWR coordinates reactivation of the relevant information distributed over multiple cortical modules [52]. SWR tends to coincide with the transition from Down to Up states of slow oscillations [53] and cortical sequence replay [8], and may contribute to the initiation of the cortical Up states [31], thus shaping a global pattern of the slow waves. Once initiated by the hippocampal input, replay in the cortical modules would be organized within patterns of sleep spindles and slow oscillation as predicted in our study.

Our study predicts importance of the N2->N3 sequence of sleep stages, however, it does not explain the role of multiple transitions between different sleep stages during night (5–6 time during normal full night sleep) or the role of REM sleep, which bounds the NREM sleep sequences. Previous studies report that REM plays important role in protecting weak memories from interference for perceptual learning [54]. We would like to speculate that the spatially localized period of synchronized alpha/theta band activity during REM may play a role similar to what we reported for localized spindles in this new study. Recent data also suggest possible role of REM in synaptic pruning [55].

### Spindles and slow oscillations serve different roles in memory consolidation

Sleep spindles are a hallmark of N2 sleep, and shown to trigger neural plasticity and to contribute to memory consolidation by promoting synaptic short- and long-term potentiation [56]. We found that sleep spindles alone were sufficient to facilitate synaptic changes in the model leading to performance improvement after the sleep. The performance gain was positively correlated with the number of sleep spindles, in agreements with human studies [21–23, 25, 26, 57]. Sleep slow oscillations are mainly observed during N3 sleep (deep sleep) and have been also associated with sleep-dependent performance enhancement. Enhancing slow oscillations by electrical stimulation improved the recall of word pairs in humans [19]. In our model, the period of sleep slow oscillations resulted in the improvement of the sequence learning task consistent with the previous experimental studies [57–59].

One key difference, however, emerged between sleep spindles and slow oscillation on the nature of the interaction between multiple memories during sleep replay. From the neuronal network perspective, the nature of this interaction could depend on the properties of the trained sequences, such as orientation and relative distance in the network space. We found that when the network was trained for two opposite (directionally) sequences, spindle activity (N2) promoted the replay of both sequences independently or with little interaction, while slow oscillations led to the competitive interaction (interference) between sequence replays. During slow oscillations (N3), when one of the memories was weak (or trained for a short time), the traveling waves driven by synaptic changes associated with the stronger memory prevented the weak memory sequence from replay and could lead to its extinction. Furthermore, the rate of synaptic changes was much faster during N3 than N2. Interestingly, when both memories were trained in the same direction, replay of the stronger sequence during slow waves could facilitate consolidation of the weaker memory, suggesting possible mechanisms for memory transfer. While we considered in our model a reduced one-dimensional network geometry, cortical traveling waves have been reported *in vivo* both for spindles [60] and for slow oscillation [61]. Importantly, while traveling slow waves in the model had global pattern and could lead to the interference between distinct memory sequences, replay was localized around the areas of awake training, so recall performance increased only for the network locations trained in awake.

The model predicts that the difference between the spatiotemporal patterns of sleep spindles vs sleep slow oscillation determined the role of spindles in minimizing interaction between memories during consolidation phase. The spindle activity was largely organized within small clusters of neurons. This allowed independent replay of many spike sequences simultaneously even when groups of neurons representing the sequences were close in space and were trained in the opposite direction. The slow oscillation was more widespread activity and showed a propagation pattern that may explain why it could lead to the competition between sequences. For two sequences trained in the opposite network direction, the order of cell firing during slow waves frequently matched the order of the strong sequence but not the weak one. Only, when both sequences were sufficiently trained in the awake state, they both mainly replayed in the correct order independently on the direction of the slow wave traveling, in agreement with experimental data [62].

This model prediction is consistent with many studies also reporting that sleep spindles emerge as local phenomena that are restricted to the specific brain regions involved in the recent task [15, 26, 63, 64]. The spatiotemporal properties of the spindle activity may depend on the underlying cortical areas [65], with local and asynchronous spindles generated in deep cortical layers by the spatially restricted core thalamocortical system, while widespread spindles reflecting the distributed matrix system [66]. Slow oscillation was shown to be a global traveling wave [61]. While coexistence of the active and silent cortical areas was reported during late sleep slow oscillation in some studies [63], this pattern was also found in our model, however, it did not prevent competition.

Although spindles often co-occur with slow oscillations [67, 68], our study is mainly focused on the differential role of sleep spindles vs slow oscillations in memory consolidation. We predict that for sleep spindles nested by the slow waves during deep sleep the outcome of consolidation would be similar to what is reported here for slow oscillation. For mixed states including the period of mainly spindle activity and some occasional slow waves, as commonly observed in humans during N2, the ratio of two will define the outcome.

During consolidation, new memory traces are stabilized or modified within the existing pool of memories [3, 5]. Memories may interfere with each other leading to forgetting [69, 70]. Such interference has been observed between [71, 72] and within memory domains [73–75]. New learning was found to be particularly vulnerable to interference when competing learning events share stimulus features and when new events are trained in short temporal succession [76, 77]. Interference may occur when one cluster of neurons “overwrites” or blocks the formation of another cluster of neurons. Sleep can protect memories from future interference [78], as well as rescue memories already damaged by interference [54, 79]. Our study predicts that sleep spindles may play a special role in protecting memories from interference, which is consistent with data of perceptual learning in humans [54]. We further predict that sleep spindles during N2 sleep and slow oscillations during N3 sleep play unique and complementary roles in the consolidation of multiple memories and the order of sleep stages—stage 2 followed by stage 3—during natural sleep is critical in preventing interference and enhancing consolidation. Our study supports a hypothesis that the basic structure of sleep stages observed repeatedly across species from low vertebrates [80] to humans [11, 12] provides an optimal environment for the consolidation of memories.

## Materials and methods

### Model description

#### Network geometry

The thalamocortical network model incorporated 100 thalamic relay (TC) and 100 reticular (RE) neurons in the thalamus, 500 pyramidal neurons (PY) and 100 inhibitory interneurons (IN) in the cortex [31, 33] organized with local synaptic connectivity (Fig 1). The PY and IN neurons received AMPA and NMDA synapses from PY neurons, and PY neurons also received GABA_A_ synapses from IN neurons. The radii of connections between cortical neurons were R_AMPA(PY-PY)_ = 5, R_NMDA(PY-PY)_ = 5, R_AMPA(PY-IN)_ = 1, R_NMDA(PY-IN)_ = 1 and R_GABAA(IN-PY)_ = 5. The TC neurons projected to RE neurons through AMPA synapses (R_AMPA(TC-RE)_ = 8), and connections from RE to TC neurons included GABA_A_ and GABA_B_ synapses (R_GABAA(RE-TC)_ = 8, R_GABAB(RE-TC)_ = 8). The radii of connections between RE and RE were R_GABAA(RE-RE)_ = 5. Thalamocortical connections were wider and mediated by AMPA synapses from TC neurons (R_AMPA(TC-PY)_ = 15, R_AMPA(TC-IN)_ = 3); corticothalamic connections were mediated by AMPA synapses from PY neurons (R_AMPA(PY-TC)_ = 10, R_AMPA(PY-RE)_ = 8). Flat connectivity profile was used for all synaptic connections. We previously tested different radii of connections and exponentially decaying profile and found qualitatively similar network dynamics, assuming that synaptic connections are scaled to maintain total synaptic input per neuron. All neurons were modeled based on the Hodgkin-Huxley kinetics. The units and description of parameters are summarized in Table 1.

**Table 1:** Main parameters. This table includes the units and description of the parameters used in the model.

Parameters	Value	Description
C_m_	1 *μ*F/cm^2^(TC;RE); 0.75 *μ*F/cm^2^(PY;IN)	Membrane capacitance
Thalamic cells		
S	2.9 × 10^−4^ cm^2^ (TC); 1.43 × 10^−4^ cm^2^ (RE)	Area of neurons
G_L_	0.01 mS/cm^2^ (TC); 0.05 mS/cm^2^ (RE)	Leakage conductance
E_L_	-70 mV (TC); -77 mV (RE)	Leakage reversal potential
G_KL_	0.024mS/cm^2^ (TC); 0.012mS/cm^2^ (RE)	Potassium leakage conductance
E_K_	-95 mV (TC; RE)	Potassium reversal potential
g_K_	10 mS/cm^2^ (RE); 12 mS/cm^2^ (TC)	Maximal potassium conductance
g_Na_	90 mS/cm^2^ (TC); 100 mS/cm^2^ (RE)	Maximal sodium conductance
g_T_	2.5 mS/cm^2^ (TC); 2.2 mS/cm^2^ (RE)	Low-threshold Ca^2+^ conductance
g_h_	0.016 mS/cm^2^ (TC); 0 mS/cm^2^ (RE)	Hyperpolarization-activated cation conductance
Cortical cells (Soma)		
S_soma_	1.0 × 10^−6^ cm^2^ (PY;IN)	Area of the axosomatic compartment
g_K_	200 mS/cm^2^ (PY;IN)	Maximal potassium conductance
g_Na_	3000 mS/cm^2^ (PY); 2500 mS/cm^2^ (IN)	Maximal sodium conductance
g_Na(p)_	15 mS/cm^2^ (PY); 0 mS/cm^2^ (IN);	Maximal persistent sodium conductance
Cortical cells (Dendrite)		
*ρ*	165 (PY); 50 (IN)	S_dend_ = *ρ* S_soma_
G_L_	0.009 mS/cm^2^ (PY); 0.009 mS/cm^2^ (IN)	Leakage conductance
E_L_	-67 mV (PY); -70 mV(IN)	Leakage reversal potential
G_KL_	0.011 mS/cm^2^ (PY); 0.009 mS/cm^2^ (IN)	Potassium leakage conductance
E_K_	-95 mV (PY;IN)	Potassium reversal potential
g_Na_	0.8 mS/cm^2^ (PY;IN)	Maximal sodium conductance
g_Na(p)_	2.5 mS/cm^2^ (PY); 0 mS/cm^2^ (IN)	Maximal persistent sodium conductance
g_HVA_	0.01 mS/cm^2^ (PY;IN)	Maximal high-threshold Ca^2+^ conductance
g_KCa_	0.05 mS/cm^2^ (PY;IN)	Slow Ca^2+^-dependent K^+^ conductance
g_Km_	0.02 mS/cm^2^ (PY); 0.015 mS/cm^2^ (IN)	Slow voltage-dependent noninactivating K^+^ conductance

#### Neuromodulators and sleep stages

The model implemented the change of neuromodulators, such as acetylcholine (ACh), histamine (HA), and GABA, in the intrinsic and synaptic currents to model transitions between sleep stages [32]. Specifically, the reduction of ACh was implemented as an increase of potassium leak conductance in TC, PY and IN neurons, a reduction of potassium leak conductance in RE cells [81], and an increase in AMPA connection strength [82]. The reduction of HA was implemented as a negative shift in the activation curve of a hyperpolarization-activated cation current (*I*_*h*_) [81, 83]. The increase of GABA was implemented as an increase of the maximal conductance of the GABAergic synapses in IN and RE neurons [32]. These synaptic and intrinsic changes were tunes to model transitions between awake state and N2 and N3 sleep stages [32].

#### Intrinsic currents: Cortex

The cortical PY and IN neurons included dendritic and axo-somatic compartments, similar to the models used in [31–33, 84, 85], that is a reduction of the multi-compartmental neuron model as described in [86]:

$$C_{m}\frac{dV_{D}}{dt}=-{ACh}_{gkl}I_{KL}-I_{Na}-I_{Na(p)}-I_{Km}-I_{KCa}-I_{HVA}-I_{L}-g(V_{D}-V_{S})-I_{syn}$$

$$0=-g(V_{S}-V_{D})-I_{Na}-I_{K}-I_{Na(p)}$$ (1)

where *C*_*m*_ is the membrane capacitance, *ACh*_*gkl*_ represents the modulation on potassium leak current *I*_*KL*_ based on the level of ACh during different sleep stages (ACh_gkl_ = 0.133, 0.228 and 0.38 for awake, N2 and N3 sleep, respectively), *I*_*Na*_ is a fast sodium current, *I*_*Na(p)*_ is a persistent sodium current, *I*_*Km*_ is a slow voltage-dependent non-inactivating potassium current, *I*_*KCa*_ is a slow Ca^2+^-dependent K^+^ current, *I*_*HVA*_ is a high-threshold Ca^2+^ current, *I*_*L*_ is the Cl^-^ leak current, g is the conductance between axo-somatic and dendritic compartment. *V*_*D*_ and *V*_*S*_ are the membrane potentials of dendritic and axosomatic compartments, and I_*syn*_ is the sum of synaptic currents to the neuron. This model was first proposed in [86] as a reduction of a multi-compartmental pyramidal cell model, based on the assumption that the current dynamics in the axosomatic compartment are fast enough to ensure that *V*_S_ is always at equilibrium state, as defined by the second equation in Eq.(1). Indeed, this reduced model has relatively high Na^+^ and K^+^ conductance values (*g*_Na_ = 3000 mS/cm^2^, *g*_K_ = 200 mS/cm^2^ [86]) in the axosomatic compartment (representing axon hillock in the model). Therefore, the full version of the axosomatic membrane voltage equation *CdVs/dt* = -*g*(*V*S−*V*_D_)–*I*_S_^int^ can be rewritten in a form ε*dVs/dt = F(Vs)*, where ε is a small parameter and *F(Vs)* represents axosomatic currents normalized to match the magnitude of the dendritic currents. Using singular perturbations analysis [87], we can find that the state variable *Vs* quickly reaches the manifold of slow motion defined by equation *F(Vs) = 0*, that corresponds to Eq (1) in our model. (See detailed discussion in [85]). The persistent sodium current *I*_*Na(p)*_ was included in the axosomatic and dendritic compartment of PY cells to increase bursting propensity. IN cells had the same intrinsic currents as those in PY except that *I*_*Na(p)*_ was not included. All the voltage-dependent ionic currents *I*_*j*_ have the similar form

$$I_{j}=g_{j}m^{M}h^{N}(V-E_{j})$$

where g_j_ is the maximal conductance, m and h are gating variables, V is the voltage of the corresponding compartment and E_j_ is the reversal potential. The dynamic of gating variables are described as

$$\frac{dx}{dt}=-\frac{x-x_{\infty }}{\tau_{x}}$$

$$\tau_{x}=(1/{(\alpha }_{x}+\beta_{x}))/Q_{T}$$

$$x_{\infty }=\alpha_{x}/{(\alpha }_{x}+\beta_{x})$$

where x = m or h. Q_T_ is a temperature related term, Q_T_ = Q^((T-23)/10)^ = 2.9529, with Q = 2.3,T = 36. The detailed description of individual currents was provided in our previous study [31].

#### Intrinsic currents: Thalamus

The thalamic TC and RE cells were modeled as a single compartment that included voltage- and calcium-dependent currents described by Hodgkin-Huxley kinetic [33]:

$$C_{m}\frac{dV}{dt}=-{ACh}_{gkl}I_{KL}-I_{Na}-I_{K}-I_{T}-I_{h}-I_{L}-I_{syn}$$

where *ACh*_*gkl*_ in TC cells is 0.4, 0.96, and 1.6 for awake, N2 and N3 sleep. *ACh*_*gkl*_ in RE cells is 0.9, 0.81, and 0.45 for awake, N2 and N3 sleep. *I*_*KL*_ is a potassium leak current, *I*_*Na*_ is a fast sodium current, *I*_*K*_ is a fast potassium current, *I*_*T*_ is a low threshold Ca^2+^ current, *I*_*h*_ is a hyperpolarization-activated cation current, *I*_*L*_ is a Cl^-^ leak current, and *I*_*syn*_ is the sum of the synaptic currents to the neuron. The hyperpolarization-activated cation current *I*_*h*_ was only included in TC neurons, not in RE neurons. The detailed description of individual currents was provided in our previous study [31]. The effect of HA on *I*_*h*_ was implemented as a shift of *HA*_*gh*_ in the activation curve [32]:

$$m_{\infty }=1/(1+exp((V+75+{HA}_{gh})/5.5))$$

where *HA*_*gh*_ is -24 mV, -2 mV, -1mV for awake, N2 and N3 sleep, respectively.

#### Synaptic currents

The equations for GABA_A_, AMPA, and NMDA synaptic currents were described by first-order activation schemes, and the GABA_B_ synaptic currents had a more complex scheme of activation that involved the activation of K^+^ channels by G proteins [88]. The equations for all synaptic currents used in this model were given in our previous studies [31, 33]. In this paper, we added the level of ACh and GABA to modulate AMPA, and GABA_A_ synaptic currents as described by

$$I_{syn}^{AMPA}={ACh}_{AMPA}g_{syn}[O](V-E_{syn})$$

$$I_{syn}^{GABA}={GABA}_{GABAA}g_{syn}[O](V-E_{syn})$$

where g_syn_ is the maximal conductance, [O] is the fraction of open channels, and E_syn_ is the reversal potential (E_AMPA_ = 0 mV, E_NMDA_ = 0 mV, and E_GABAA_ = -70 mV). ACh_AMPA_ is the variable that modulates AMPA synaptic currents for cortical PY-PY, TC-PY, and TC-IN connections by the level of ACh. ACh_AMPA_ from PY cells is 0.133, 0.1938, and 0.4332 for awake, N2 and N3 sleep. ACh_AMPA_ from TC cells is 0.6, 0.72 and 1.2 for awake, N2 and N3 sleep. GABA_GABAA_ is the variable that modulates GABA synaptic currents for cortical IN-PY, RE-RE and RE-TC connections. GABA_GABAA_ from IN cells is 0.22, 0.264 and 0.44 for awake, N2 and N3 sleep. GABA_GABAA_ from RE cells is 0.6, 0.72 and 1.2 for awake, N2 and N3 sleep, respectively.

The maximal conductance for each specific synapse was g_GABAA(RE-TC)_ = 0.06 μS, g_GABAB(RE-TC)_ = 0.0025 μS, g_GABAA(RE-RE)_ = 0.1μS, g_AMPA(TC-RE)_ = 0.06 μS, g_AMPA(TC-PY)_ = 0.14 μS, g_AMPA(TC-IN)_ = 0.12 μS, g_AMPA(PY-PY)_ = 0.24 μS, g_NMDA(PY-PY)_ = 0.01 μS, g_AMPA (PY-IN)_ = 0.12 μS, g_NMDA(PY-IN)_ = 0.01 μS., g_AMPA (PY-TC)_ = 0.04 μS, g_AMPA (PY-RE)_ = 0.08 μS and g_GABAA(IN-PY)_ = 0.24 μS.

In addition, spontaneous miniature EPSPs and IPSPs were implemented for PY-PY, PY-IN and IN-PY connections. The arrival times of spontaneous miniature EPSPs and IPSPs were modeled by Poisson processes [89], with time-dependent mean rate μ = (2/(1+exp(-(t-t_0_)/F))-1)/250[33], where t_0_ is a time instant of the last presynaptic spike [84]. The mEPSP frequency (F) and amplitude (A) were F_PY-PY_ = 30, F_PY-IN_ = 30, F_IN-PY_ = 30, A_PY-PY_ = 0.2 mV, A_PY-IN_ = 0.2 mV, and A_IN-PY_ = 0.2 mV.

#### Spike-timing dependent synaptic plasticity (STDP)

Facilitation or depression of the synaptic strength is believed to underlie learning in the brain. Here we used STDP model of synaptic plasticity to adjust the synaptic connections between cortical pyramidal neurons based on the relative timing of the pre- and postsynaptic spikes. The change of excitatory synaptic connections (g_AMPA_) and the amplitude of mEPSC (A_mEPSC_) were described as in our previous paper [31]:

$$g_{AMPA}\leftarrow g_{AMPA}+g_{max}F(\Delta t)$$

$$A_{mEPSC}\leftarrow A_{mEPSC}+fA_{PY-PY}F(\Delta t)$$

where g_max_ is the maximal synaptic conductance of g_AMPA_. f = 0.01 is a factor representing the change of STDP on A_mEPSC_ is slower than on g_AMPA_. F is the STDP function that shows the change of synaptic connections as a function of the relative timing (Δt) of pre- and postsynaptic spikes [90],

$$F(\Delta t)=\left\{ \begin{array}{c} A_{+}e^{-\frac{\left|\Delta t\right|}{t_{+}}},if\;\Delta t>0\\ {-A}_{-}e^{-\frac{\left|\Delta t\right|}{t_{-}}},if\;\Delta t<0 \end{array}\right.$$

where parameters A_+_ and A_-_ determine the maximum amounts of synaptic modification. Here, we set A_+_ = A_-_ = 0.002, and *τ*_+_ = *τ*_-_ = 20 ms. We reduced the STDP amplitude A_+_ and A_-_ to 0.001 during slow-wave sleep to account for reduction of ACh [91]. We assumed that the synaptic efficacy should stay within [0, 200%] range of the initial synaptic weights to prevent STDP from runaway synaptic dynamics. We would like to note that in vivo the rate of synaptic potentiation is slower than that in the model and typically saturates around 150% of cortical neurons over a full night [92]. Because of that, although our simulation times (in absolute unites) are much shorter than a full night, the change of the synaptic weights in the trained region was sufficient to observe the performance improvement after sleep.

#### Training and test

For most of the simulations, training pattern included 5 groups of neurons that were activated in sequential order in space and time, with 5 msec delay between subsequent groups activation. Each group was a set of 5 adjacent neurons drawn from a contiguous 25 cell subregion of the full 500 cell network. For example, if the sequence started at neuron #200, these 5 groups were: A(#200–204), B(#205–209), C(#210–214), D(#215–219),E(#220–224). Each group was stimulated for 10 ms. Thus during training, the neuronal activity in these groups reflected the order of the trained sequence, e.g., “ABCDE”. During test sessions, the model was only presented with the first input at group “A” to recall the trained sequence “ABCDE” within a 350ms response window. During both training and test sessions, each trial was repeated every 1s. To test pattern completion outside trained area, we selected a random location (e.g., A_i_, i = 1,2,.., N) and tested for virtual sequences (A_i_→B_i_→C_i_→D_i_→E_i_) that were defined to have similar adjacency constraints (A_i_ is next to B_i_, which is next to C_i_, etc.) as for the actual trained sequence (A→B→C→D→E). To test “non-linear” training patterns, we used smaller groups of neurons (each group was a set of 2 adjacent neurons drawn from a contiguous 10 cell subregion) to ensure that non-adjacent groups are synaptically connected. For example, if the sequence started at neuron #200, the groups were: A(#200–201), B(#202–203), C(#204–205), D(#206–207), E(#208–209). During training, these groups were activated in “non-linear” order (A→C→B→D→E) with the same time delay 5msec between subsequent groups activation.

### Statistical analysis

When data were normally distributed based on statistical test, the numerical values are given as mean ± SEM, where SEM is standard error of the mean. Otherwise, we used median ± interquartile range (IQR) to report the data. For each experiment, 10 simulations with different random seeds were performed. Data were first tested for normal distribution by the Anderson-Darling test, and if data had a normal distribution, the parametric test was used; otherwise, the equivalent nonparametric test was applied. If only two groups of data were compared, the two-sample t-test (parametric) or the Mann–Whitney U test (nonparametric) was used. When data were paired, nonparametric Wilcoxon signed rank test was used. When more than two groups of data were compared, One-way ANOVA (parametric) or Kruskal-Wallis ANOVA test (nonparametric) with Bonferroni’s post hoc test was applied. To compare the means of two or more columns and two or more rows of the observations, two-way ANOVA was used.

### Data analysis

#### Sequence learning analysis

To model sequence learning, the model was presented with multiple trials of sequential input to the groups of selected cortical neurons. The performance of sequence recall was measured by the percentage of success of sequence recall during test sessions when only the first group of a sequence was stimulated. First, we detected the network sequence using the following steps: 1) We detected all spikes for five groups of neurons (each group contains five neurons) within a 350ms response time window (starting from the time when test stimulus was applied); 2) We smoothed the firing rate of each group by convoluting the average instantaneous firing rate of five neurons with a Gaussian kernel (50ms window size); 3) The firing sequence of the groups was determined by ordering the peaks of their smoothed firing rates during 350ms window. Next, we applied a String Match (SM) method to measure the similarity between each detected sequence and an ideal sequence (e.g. S = “ABCDE”). The SM was calculated as $SM=2*N-\sum_{i=1}^{N}\left|L(S_{1},S_{2}[i])-i\right|$, where *S*_1_ is the test sequence generated by the network, *S*_2_ is the subset of ideal sequence that only contains the same elements as *S*_1_, *N* is the sequence length of S_1_, *L*(*S*_1_,*S*_2_[*i*]) represents the location of element S_2_[*i*] in a sequence *S*_1_. SM was then normalized by dividing by M, where M is two times the length of S. For example, if the ideal sequence S was “ABCDE” and S_1_ was”ACDB”, then S_2_ =“ABCD”, N = 4. The location of element ‘A’ in S_1_ is L(S_1_, ‘A’) = 1. ‘B’ in S_1_ is L(S_1_, ‘B’) = 4, ‘C’ in S_1_ is L(S_1_, ‘C’) = 2, ‘D’ in S_1_ is L(S_1_, ‘D’) = 3. Therefore, SM = 2*4- (|1–1|+|4–2|+|2–3|+|3–4|) = 4. After SM was normalized by M = 10, it became 0.4, indicating the recalled sequence has 40% similarity to the ideal sequence. If the ideal sequence S was “ABCDE” and S_1_ was”ABCDE”, then S_2_ =“ABCDE”, N = 5 and SM = 2*5–0 = 10, or 1.0 after normalization by 10. The performance was calculated as the percentage of recalled sequences with SM≥Th during the test session. In this paper, we selected a threshold of Th = 0.8, indicating a recalled sequence with at least 80% similarity to the ideal sequence was counted as a successful recall. Baseline performance (before training) of the network was around 15% for Th = 0.8 due to the random spiking. If higher threshold Th = 1.0 was selected, the baseline performance became almost zero.

#### Sequence replay measurement during sleep

The replay measure was calculated using SM method, similar to above: $SM=2*N-\sum_{i=1}^{N}\left|L(S,S_{ideal}[i])-i\right|$, where *S*_ideal_ stands for correct sequence. The sequence S was defined with the following steps: (i) First, we identified all spike times $t_{n}^{m}$ (here *m* stands for spike number, *n* stands for the neuron index) of the first neuron (with index *n*) in the area of interest (*e*.*g*., *n* = 200). For each spike time $t_{n}^{m}$ (e.g. n = 200, m = 1,…,M, where M is a total number of spikes) we repeated steps (ii)-(v) as follows: (ii) Given one particular spike $t_{n}^{m_{0}}$ in a leading neuron, we identified the closest in time spike in the next neuron with index *n+1*, i.e. found a spike $t_{n+1}^{m_{1}}$ for which | $t_{n}^{m_{0}}‑t_{n+1}^{m_{1}}$| is minimal. If the spike time difference exceeded threshold | $t_{n}^{m_{0}}‑t_{n+1}^{m_{1}}$|>50 ms, the spike $t_{n+1}^{m_{1}}$ was rejected from consideration (iii). The step (ii) was subsequently repeated for all other neurons *n+2*, *n+3*, *n+4*, *…*, *n+25*. E.g. for neuron *n+2* we identified the closest spike $t_{n+2}^{m_{2}}$ to spike time $t_{n+1}^{m_{1}}$. If $t_{n+1}^{m_{1}}$ was rejected based on threshold criteria, then the spike time $t_{n}^{m_{0}}$ was used instead. (iv) For each group, we identified average firing times as $T_{i}=\frac{1}{5}\sum_{j=n+5i}^{n+5(i+1)}t_{j}^{m_{j}}$; (v) The sequence S was formed according to the order of average firing times *T*_*i*_.

#### Analysis of synaptic weights

Synaptic weights between neurons in a direction of sequence activation were enhanced due to the sequence replay. The mean of the changes of synaptic weights associated with a given sequence was used to characterize memory strength. The probability of enhancing Seq2 in two-sequence learning was calculated by counting the relative (over the total number of trials) number of trials that had a trend of increasing the mean synaptic weights associated with Seq2 for the last 100s of N3 sleep.

#### Spatiotemporal pattern analysis

The spatial correction was calculated as follows: 1) The spike train of each neuron was convoluted with a Gaussian function (*window* = 1000*ms*, *with μ* = 500*ms*, *σ* = 5*ms*); 2) Within each spindle or Up state of slow oscillation, the cross correlation of the convoluted spike trains for each pair of neurons was calculated; 3) The averaged peak of the cross-correlation function was assessed for varying distances between network sites. For example, for the distance of 2, we averaged the peaks of the cross-correlation function from all the possible pairs of neurons at distance 2 (# 1 and #3, #2 and #4, #3 and #5, etc.). To detect the spatiotemporal cluster, the following steps were applied: 1) the spatiotemporal activity of spindle or slow oscillation was smoothed by a 2D Gaussian kernel (using **imfilter** function in MATLAB); 2) Different contiguous activation regions were labeled based on connected components of smoothed spatiotemporal pattern (using **bwlabel** function in MATLAB). The region with the same-labeled number was considered as one cluster. The histogram of neuron number within each cluster was plotted during spindle and slow oscillations.

#### Computational methods

All model simulations were performed using a fourth-order Runge-Kutta integration method with a time step of 0.02 ms. Source C++ was compiled on a Linux server using the g++ compiler. Part of the simulation was run on the Neuroscience Gateway[93]. All data processing was done with custom-written programs in Matlab (MathWorks, Natick, MA).

## Supporting information

S1 Fig The pattern of slow oscillations during N3 sleep after the sequence ABCDE was trained. *Top*, A characteristic example of single cell activity. *Top Middle*, Characteristic example of the network dynamics. Membrane voltage of pyramidal neurons is indicated with a color code; white stars indicate the site of Up-state initiation. *Bottom Middle*, Up-state initiation sites over the entire simulation time are indicated by black dots. Bottom, the probability of local Up-state initiation over the entire network.
(TIFF)

S2 Fig Performance change for untrained areas of the network. **a**) The sequence learning paradigm: awake state, N2, N3 sleep, awake state. **b**) The expanded view of characteristic spatiotemporal patterns during three typical samples of training and test sessions. The “ABCDE” is the trained region. The A_1_ (#100–104), A(#200–204) and A_3_(#300–304) are the neurons that were stimulated during test sessions. Note pattern completion after the sleep for trained sequence ABCDE but not for untrained sequences starting at A1 or A3. **c**) The difference in performance improvement (after sleep test session minus before training test session) for multiple un-trained sequences. The performance was tested by applying test stimulation to random neurons outside the trained region. Star–the performance improvement of sequence recall was tested in direction of the cell indices increase from the test neurons; Circle–the performance improvement of sequence recall was tested in the opposite direction. In both cases, the algorithm attempted to detect any sequence in the defined direction. **d**) The histogram combining all data of performance improvement for all un-trained sequences (random locations outside network area 200–225). **e**) Effect of training on sequence replay. The difference between the normalized counts of direct and reverse sequence replays calculated for different network locations. For each location/direction we calculated the total number of the sequence replays in the trained network (F) and normalized it by that in untrained network (F0). Black line represents mean, and the grey patch error bar represents SEM.
(TIFF)

S3 Fig NREM sleep improves the performance completion of the complex sequence: ACBDE. Location of neuronal groups A-E were: A(#200–201), B(#202–203),C(#204–205),D(#206–207),E(#208–209). **a**) The change of synaptic connectivity matrix after training (*left*) and after sleep (*right*). **b**) The performance of ACBDE in test sessions. Data were analyzed using one-way ANOVA with Bonferroni’s post hoc test. * p<0.05, ** p<0.01, *** p<0.001. N.S. represents no significant difference.
(TIFF)

S4 Fig The characteristic of awake activity in memory consolidation. **a**) The sequence learning paradigm. The network was kept awake. The expanded view of characteristic spatiotemporal patterns (*top*), LFP (*middle top*), single cell activity of neuron #200 (*middle bottom*), and performance during test sessions (*bottom*) when awake firing rate was around 0.6Hz (**b**) and awake firing rate was increased to 1.7Hz (**c**). Data were analyzed using two-sample t test. * p<0.05, ** p<0.01, *** p<0.001. N.S. represents no significant difference.
(TIFF)

S5 Fig The characteristic of spindle activity in memory consolidation. **a**) The sequence learning paradigm. The cortical network activity during transitions from the awake state to N2 sleep and back to the awake state. The expanded view of characteristic spatiotemporal patterns (*top*), LFP (*middle top*), single cell activity of neuron #200 (*middle bottom*), and performance during test sessions (*bottom*) when spindle density was around 5/min (**b**) and spindle density was reduced to around 3/min (**c**). Data were analyzed using two-sample t test. * p<0.05, ** p<0.01, *** p<0.001. N.S. represents no significant difference.
(TIFF)

S6 Fig The characteristic of slow oscillation in memory consolidation. **a**) The sequence learning paradigm. The cortical network activity during transitions from the awake state to N3 sleep and back to the awake state. The expanded view of characteristic spatiotemporal patterns (top), LFP (middle top), single cell activity of neuron #200 (middle bottom), and performance during test sessions (bottom) when the frequency of slow oscillations was around 0.7Hz (**b**) and the frequency of slow oscillations was reduced to around 0.3Hz (**c**). Data were analyzed using two-sample t test. * p<0.05, ** p<0.01, *** p<0.001. N.S. represents no significant difference.
(TIFF)

S7 Fig The effects of distance and orientation of training between two sequences. The difference (Y-axis) between accumulated synaptic changes for the Seq2 in presence of Seq 1 vs when the Seq 2 was presented alone, for different distances between two sequences (X-axis). N3 sleep (**a**) and N2 sleep (**b**). Zero synaptic difference indicates no interaction between sequences during consolidation. Solid lines are for Seq2 having the opposite direction of training compare to Seq1; dotted lines are for Seq2 trained in the same direction as the Seq1.
(TIFF)
