# Supplementary figures and images for: Differential roles of sleep spindles and sleep slow oscillations in memory consolidation
Source: PLoS Comput Biol. 2018 Jul 9;14(7):e1006322. doi: 10.1371/journal.pcbi.1006322 (PMC6053241; doi:10.1371/journal.pcbi.1006322)

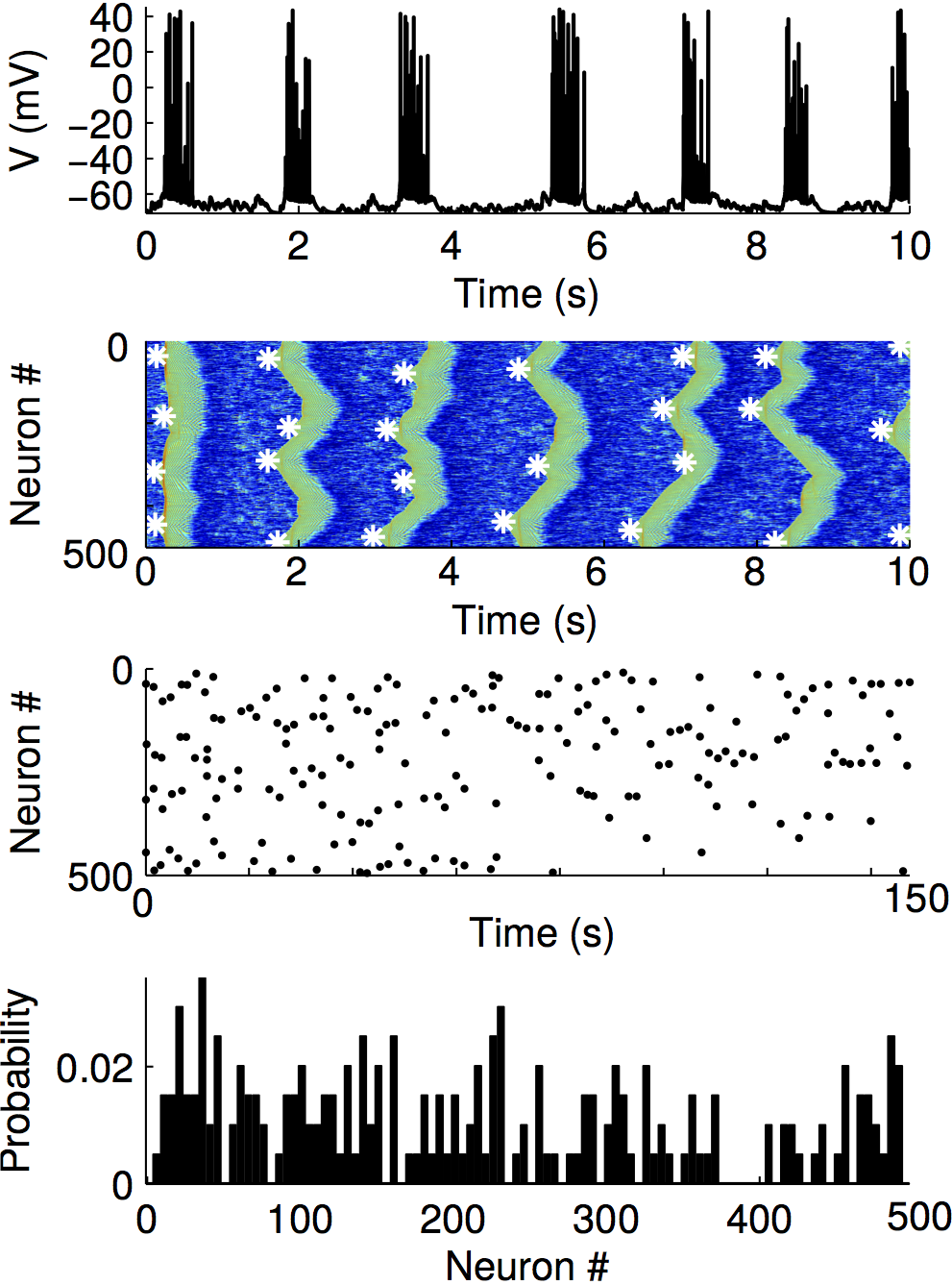

Supplement: S1 Fig — Top, A characteristic example of single cell activity. Top Middle, Characteristic example of the network dynamics. Membrane voltage of pyramidal neurons is indicated with a color code; white stars indicate the site of Up-state initiation. Bottom Middle, Up-state initiation sites over the entire simulation time are indicated by black dots. Bottom, the probability of local Up-state initiation over the entire network. (TIFF) [file pcbi.1006322.s001.tiff]

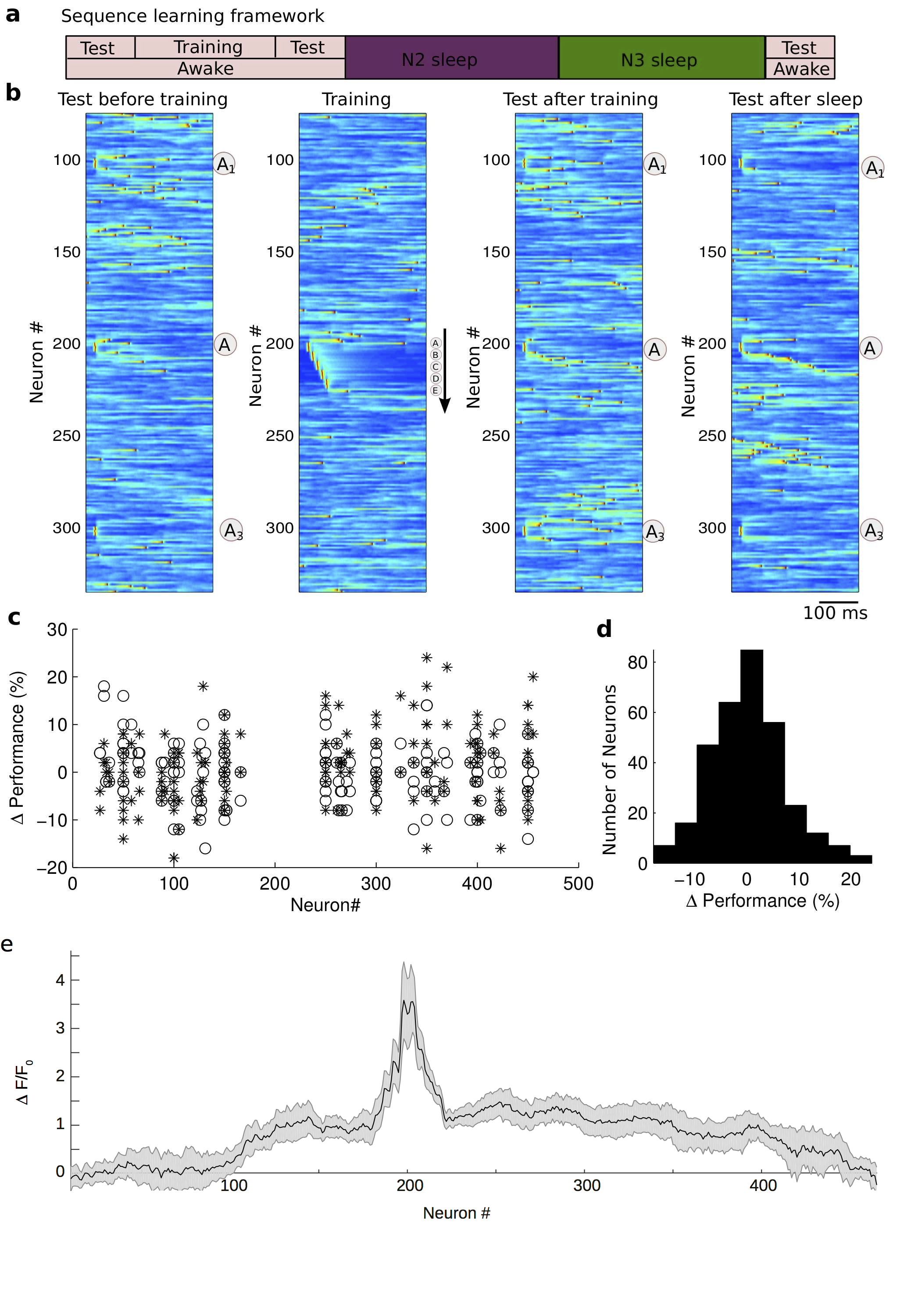

Supplement: S2 Fig — a) The sequence learning paradigm: awake state, N2, N3 sleep, awake state. b) The expanded view of characteristic spatiotemporal patterns during three typical samples of training and test sessions. The “ABCDE” is the trained region. The A1 (#100–104), A(#200–204) and A3(#300–304) are the neurons that were stimulated during test sessions. Note pattern completion after the sleep for trained sequence ABCDE but not for untrained sequences starting at A1 or A3. c) The difference in performance improvement (after sleep test session minus before training test session) for multiple un-trained sequences. The performance was tested by applying test stimulation to random neurons outside the trained region. Star–the performance improvement of sequence recall was tested in direction of the cell indices increase from the test neurons; Circle–the performance improvement of sequence recall was tested in the opposite direction. In both cases, the algorithm attempted to detect any sequence in the defined direction. d) The histogram combining all data of performance improvement for all un-trained sequences (random locations outside network area 200–225). e) Effect of training on sequence replay. The difference between the normalized counts of direct and reverse sequence replays calculated for different network locations. For each location/direction we calculated the total number of the sequence replays in the trained network (F) and normalized it by that in untrained network (F0). Black line represents mean, and the grey patch error bar represents SEM. (TIFF) [file pcbi.1006322.s002.tiff]

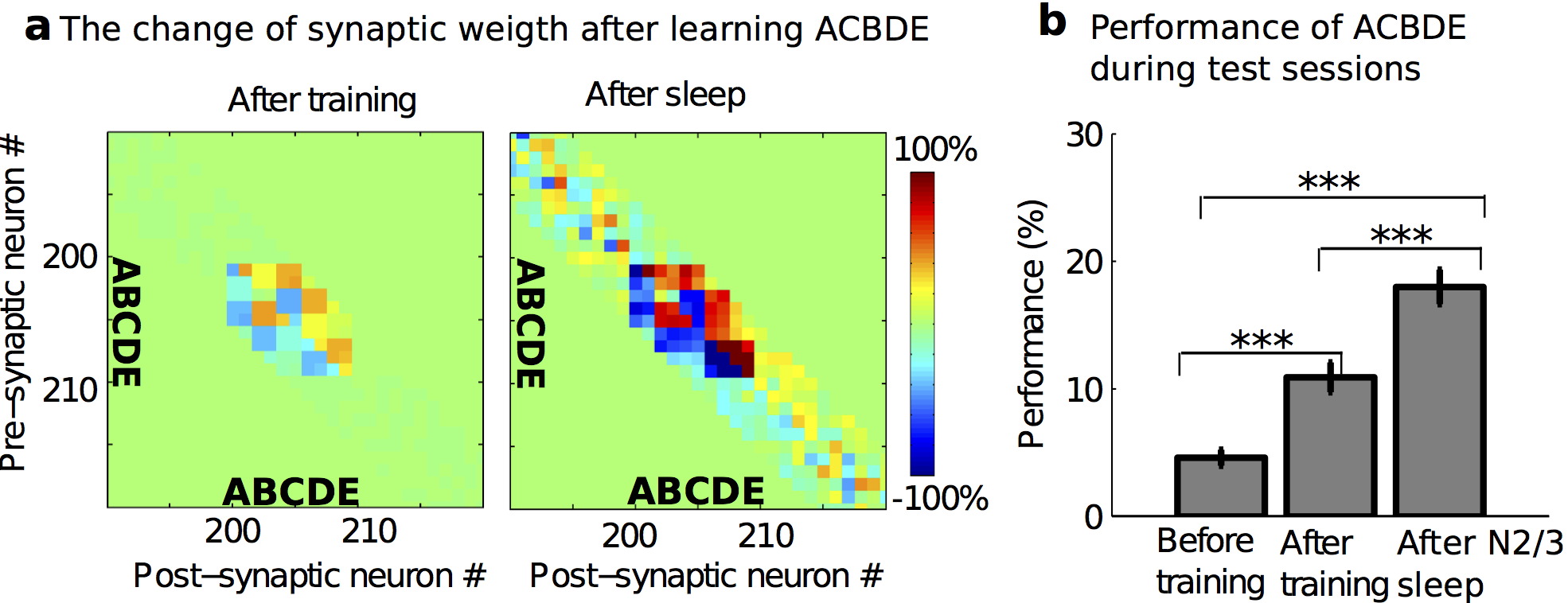

Supplement: S3 Fig — Location of neuronal groups A-E were: A(#200–201), B(#202–203),C(#204–205),D(#206–207),E(#208–209). a) The change of synaptic connectivity matrix after training (left) and after sleep (right). b) The performance of ACBDE in test sessions. Data were analyzed using one-way ANOVA with Bonferroni’s post hoc test. * p<0.05, ** p<0.01, *** p<0.001. N.S. represents no significant difference. (TIFF) [file pcbi.1006322.s003.tiff]

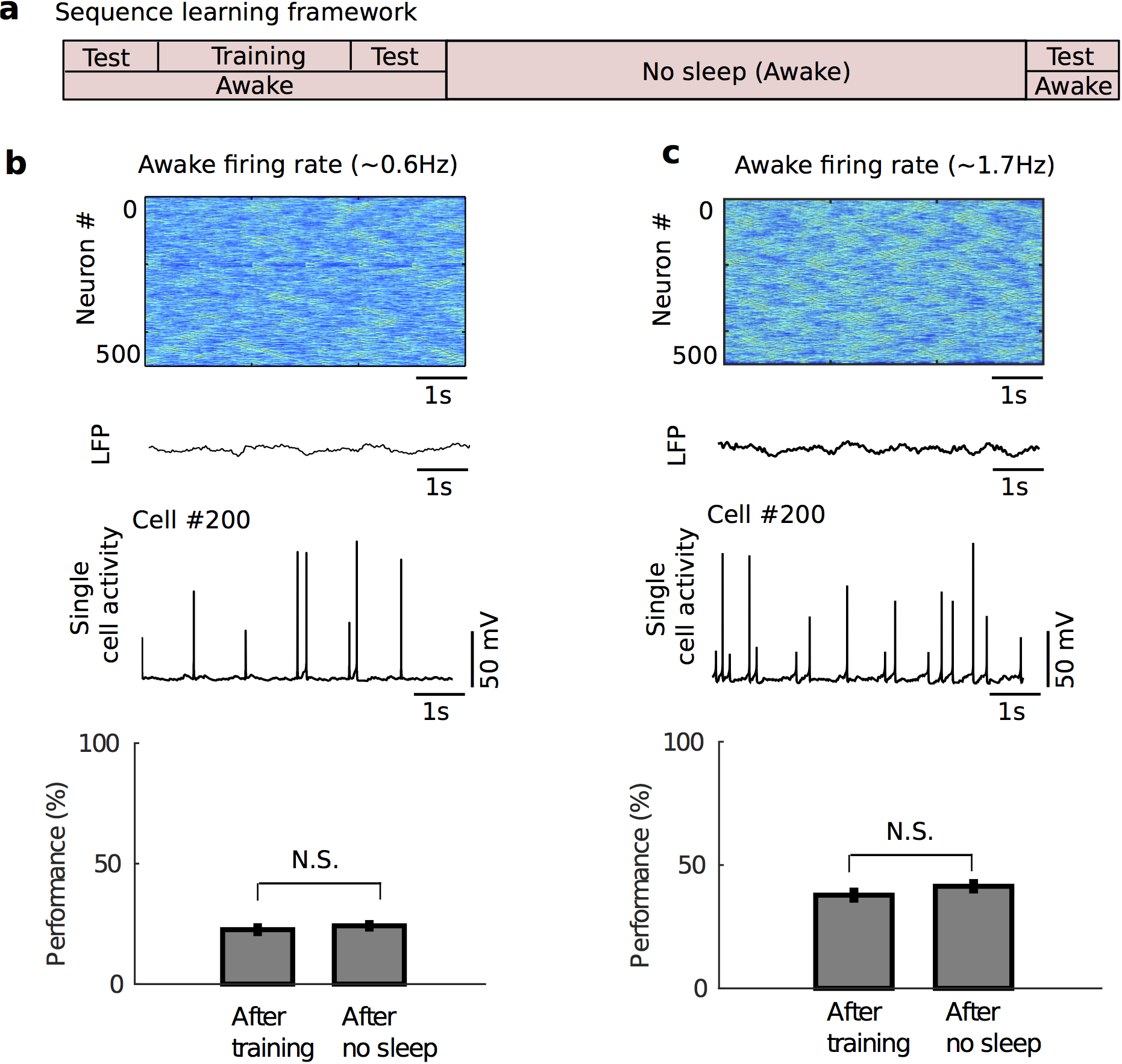

Supplement: S4 Fig — a) The sequence learning paradigm. The network was kept awake. The expanded view of characteristic spatiotemporal patterns (top), LFP (middle top), single cell activity of neuron #200 (middle bottom), and performance during test sessions (bottom) when awake firing rate was around 0.6Hz (b) and awake firing rate was increased to 1.7Hz (c). Data were analyzed using two-sample t test. * p<0.05, ** p<0.01, *** p<0.001. N.S. represents no significant difference. (TIFF) [file pcbi.1006322.s004.tiff]

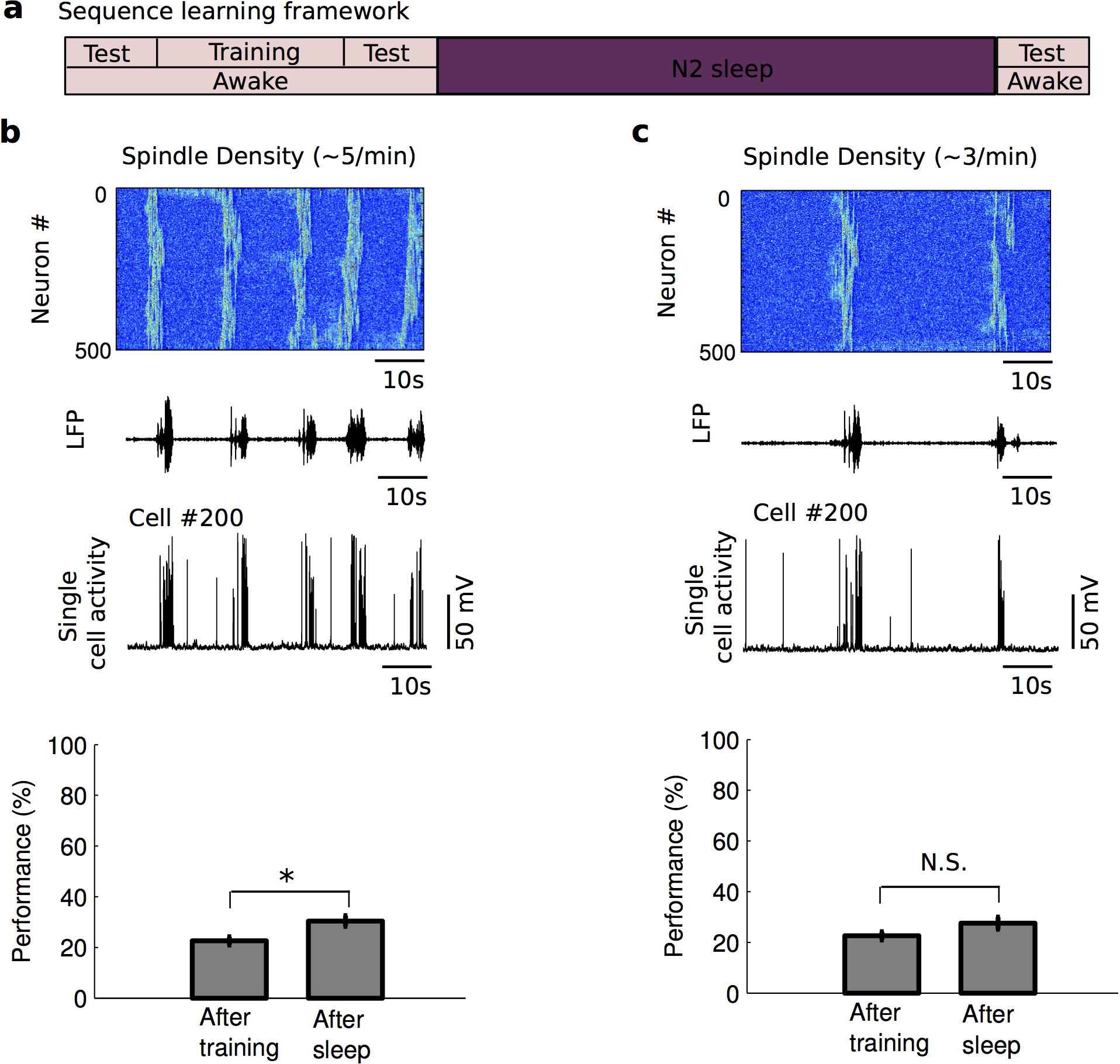

Supplement: S5 Fig — a) The sequence learning paradigm. The cortical network activity during transitions from the awake state to N2 sleep and back to the awake state. The expanded view of characteristic spatiotemporal patterns (top), LFP (middle top), single cell activity of neuron #200 (middle bottom), and performance during test sessions (bottom) when spindle density was around 5/min (b) and spindle density was reduced to around 3/min (c). Data were analyzed using two-sample t test. * p<0.05, ** p<0.01, *** p<0.001. N.S. represents no significant difference. (TIFF) [file pcbi.1006322.s005.tiff]

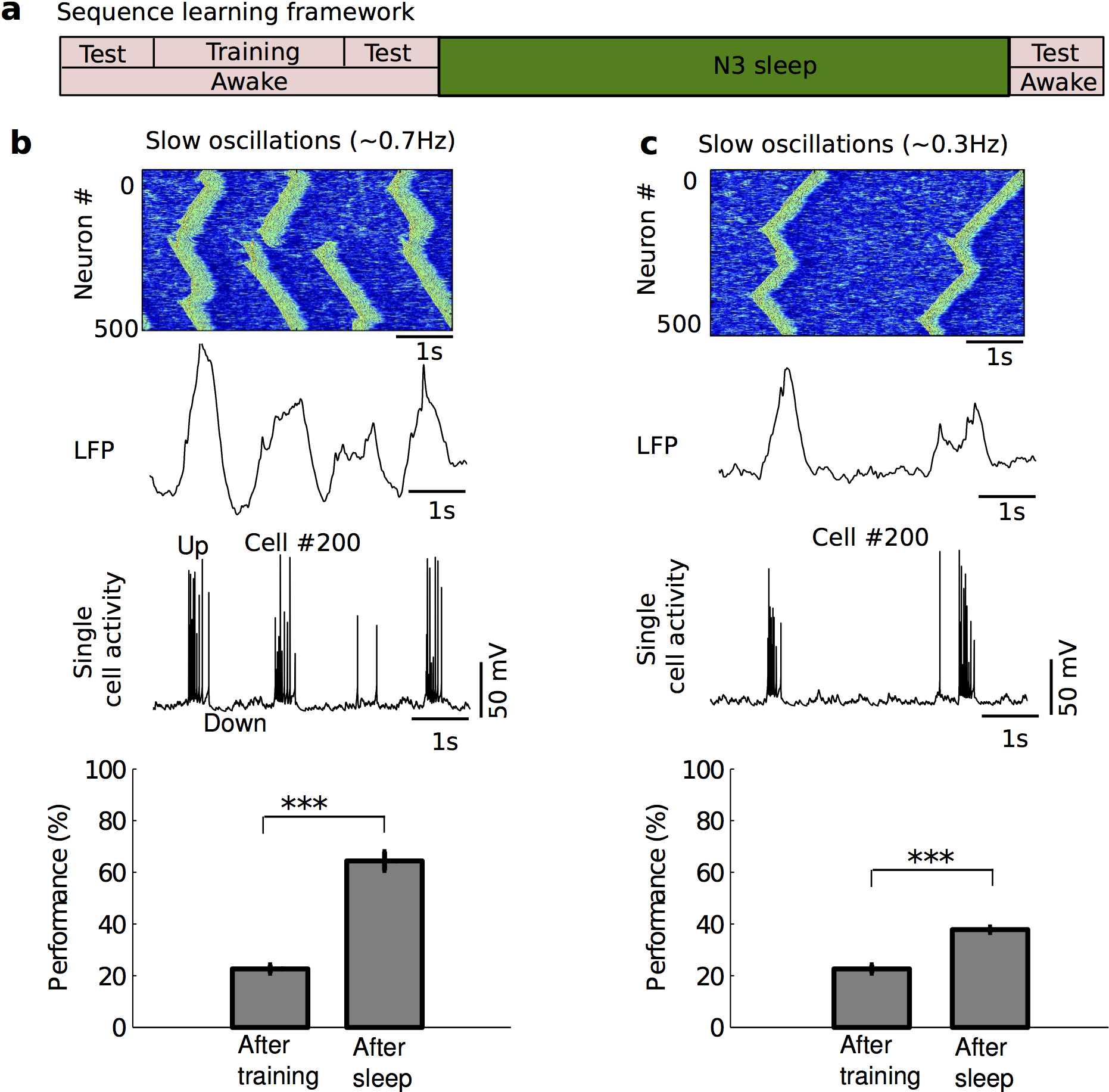

Supplement: S6 Fig — a) The sequence learning paradigm. The cortical network activity during transitions from the awake state to N3 sleep and back to the awake state. The expanded view of characteristic spatiotemporal patterns (top), LFP (middle top), single cell activity of neuron #200 (middle bottom), and performance during test sessions (bottom) when the frequency of slow oscillations was around 0.7Hz (b) and the frequency of slow oscillations was reduced to around 0.3Hz (c). Data were analyzed using two-sample t test. * p<0.05, ** p<0.01, *** p<0.001. N.S. represents no significant difference. (TIFF) [file pcbi.1006322.s006.tiff]

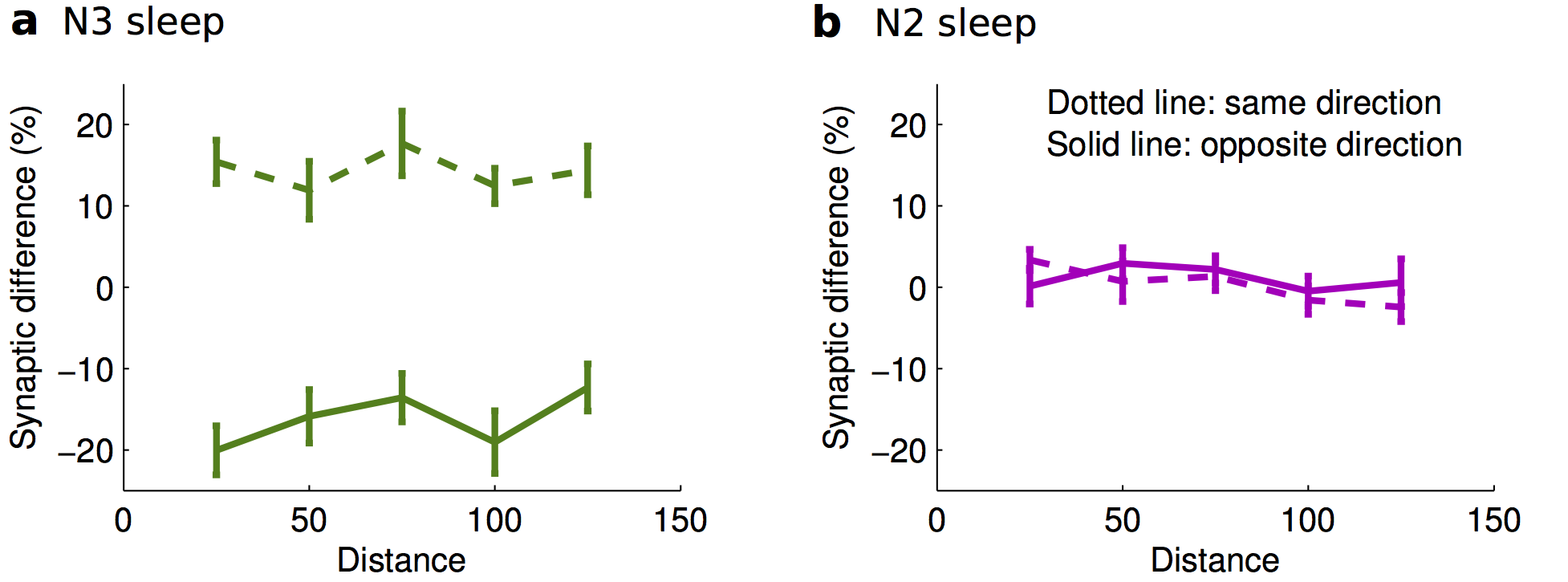

Supplement: S7 Fig — The difference (Y-axis) between accumulated synaptic changes for the Seq2 in presence of Seq 1 vs when the Seq 2 was presented alone, for different distances between two sequences (X-axis). N3 sleep (a) and N2 sleep (b). Zero synaptic difference indicates no interaction between sequences during consolidation. Solid lines are for Seq2 having the opposite direction of training compare to Seq1; dotted lines are for Seq2 trained in the same direction as the Seq1. (TIFF) [file pcbi.1006322.s007.tiff]
